# The Matrix Protein of Nipah Virus Targets the E3-Ubiquitin Ligase TRIM6 to Inhibit the IKKε Kinase-Mediated Type-I IFN Antiviral Response

**DOI:** 10.1371/journal.ppat.1005880

**Published:** 2016-09-13

**Authors:** Preeti Bharaj, Yao E. Wang, Brian E. Dawes, Tatyana E. Yun, Arnold Park, Benjamin Yen, Christopher F. Basler, Alexander N. Freiberg, Benhur Lee, Ricardo Rajsbaum

**Affiliations:** 1 Department of Microbiology and Immunology, University of Texas Medical Branch, Gavelston, Texas, United States of America; 2 Department of Microbiology, Icahn School of Medicine at Mount Sinai, New York, New York, United States of America; 3 Department of Pathology University of Texas Medical Branch, Galveston, Texas, United States of America; Harvard Medical School, UNITED STATES

## Abstract

For efficient replication, viruses have developed mechanisms to evade innate immune responses, including the antiviral type-I interferon (IFN-I) system. Nipah virus (NiV), a highly pathogenic member of the *Paramyxoviridae* family (genus *Henipavirus*), is known to encode for four P gene-derived viral proteins (P/C/W/V) with IFN-I antagonist functions. Here we report that NiV matrix protein (NiV-M), which is important for virus assembly and budding, can also inhibit IFN-I responses. IFN-I production requires activation of multiple signaling components including the IκB kinase epsilon (IKKε). We previously showed that the E3-ubiquitin ligase TRIM6 catalyzes the synthesis of unanchored K48-linked polyubiquitin chains, which are not covalently attached to any protein, and activate IKKε for induction of IFN-I mediated antiviral responses. Using co-immunoprecipitation assays and confocal microscopy we show here that the NiV-M protein interacts with TRIM6 and promotes TRIM6 degradation. Consequently, NiV-M expression results in reduced levels of unanchored K48-linked polyubiquitin chains associated with IKKε leading to impaired IKKε oligomerization, IKKε autophosphorylation and reduced IFN-mediated responses. This IFN antagonist function of NiV-M requires a conserved lysine residue (K258) in the bipartite nuclear localization signal that is found in divergent henipaviruses. Consistent with this, the matrix proteins of Ghana, Hendra and Cedar viruses were also able to inhibit IFNβ induction. Live NiV infection, but not a recombinant NiV lacking the M protein, reduced the levels of endogenous TRIM6 protein expression. To our knowledge, matrix proteins of paramyxoviruses have never been reported to be involved in innate immune antagonism. We report here a novel mechanism of viral innate immune evasion by targeting TRIM6, IKKε and unanchored polyubiquitin chains. These findings expand the universe of viral IFN antagonism strategies and provide a new potential target for development of therapeutic interventions against NiV infections.

## Author Summary

Nipah virus (NiV) is a zoonotic paramyxovirus causing severe respiratory and encephalitic illness with case fatality rates of 40 to 90%. The host type-I interferon (IFN-I) system protects against viral infections; however, to establish productive infection NiV has developed mechanisms to evade these host antiviral responses. An important component of the IFN system is the IKKε kinase, which is directly involved in IFN-I production and IFN-I signaling. The activity of the IKKε kinase is regulated by unanchored K48-linked polyubiquitin chains, a novel form of ubiquitin that is not covalently attached to any protein and can induce activation of kinases by promoting protein oligomerization. These unanchored polyubiquitin chains that activate IKKε are generated by the E3-ubiquitin ligase TRIM6. Here we demonstrate that the matrix structural protein (M) of NiV, which is important for virus assembly and budding, also has IFN-I antagonist functions and interferes with the host antiviral response. We found that NiV-M interacts with TRIM6 and promotes its degradation. Consequently, association of unanchored polyubiquitin chains with IKKε is reduced leading to impaired IKKε activation and ineffective IFN responses. Since the matrix protein is present in the virions and is released immediately after virus entry into the cell, this provides an efficient mechanism to escape the host antiviral response. These data may help explain the highly pathogenic potential of these viruses.

## Introduction

Innate immune responses are initiated when conserved features of microbial pathogens referred to as pathogen-associated molecular patterns (PAMPs) are recognized by host pattern-recognition receptors (PRRs), such as Toll-like receptors (TLRs) and retinoic acid-inducible gene I (RIG-I)-like receptors (RLRs) [1, 2]. During viral infections, single or double-stranded RNAs generated by viruses can be recognized by the endosomal TLR3 or the cytoplasmic RIG-I/Melanoma Differentiation-Associated gene 5 (MDA-5) [3, 4]. When activated, TLR3 signals through the adaptor protein Toll-IL-1R (TIR) domain-containing adaptor-inducing IFNβ (TRIF). On the other hand, RIG-I and MDA-5 utilize the adaptor protein MAVS localized in the mitochondrial membrane. TLR and RLR signaling requires activation of multiple signaling components converging at the level of the serine/threonine kinases TANK binding kinase-1 (TBK1) and IκB kinase-ε (IKKε) [5, 6], which phosphorylate the transcription factors IFN regulatory factor 3 (IRF3) and IRF7 [7, 8]. This promotes the nuclear accumulation of IRF3 and IRF7, triggering the expression of type-I IFNs (IFN-I) [3, 4, 6]. Pathogen recognition by TLRs and RLRs also results in activation of the nuclear factor kappa-light-chain-enhancer of activated B cells (NF-κB), which promotes the induction of various chemokines and cytokines including interleukin-6 (IL-6) and tumor necrosis factor (TNFα) [9, 10]. NF-κB is also important for the optimal production of IFN-I [5–8]. Secreted IFN-I bind to the heterodimeric type I IFN receptor (IFNAR), thus activating the (Janus kinase) JAK1 and TYK2 kinases, which phosphorylate the transcription factors STAT1 (signal transducer and activator of transcription) and STAT2. Together STAT1 and STAT2 with IRF9 form the IFN stimulated gene factor 3 (ISGF3) that translocates to the nucleus for induction of numerous IFN-stimulated genes (ISGs), triggering an antiviral state [11]. Importantly, formation of the ISGF3 complex and induction of the full breadth of ISGs also requires STAT1 phosphorylation by IKKε, which plays a non-redundant role in the IFN signaling pathway [12–14].

The Tripartite Motif (TRIM) family of proteins constitutes over 70 proteins, which are characterized by the presence of a RING, B box, and a coiled-coil domain (collectively called RBCC), has been implicated in innate immune signaling pathways by acting as E3-Ubiquitin ligases [15–19]. Recently, we have shown that an unprecedented large number of TRIMs positively regulates innate immune responses [19, 20]. In-depth molecular characterization focused on TRIM6, which we showed is important in both IFN-I production and IFN-I signaling pathways. Specifically, TRIM6 together with the E2-conjugase UbE2K synthesize unanchored K48-linked polyubiquitin chains that activate IKKε, culminating in the induction of a subset of ISGs essential for the antiviral response [13].

To successfully replicate in a host cell, viruses have devised mechanisms to evade the host IFN response by inhibiting key components of the pathway leading to either reduced IFN production or reduced ISG induction. Some members of the family *Paramyxoviridae* have a RNA-editing mechanism to produce alternative proteins from the P gene, namely V and W, that have been shown to have IFN antagonistic activities [21]. For example, the rubulavirus V proteins target STATs for proteasome-mediated degradation [22, 23]; the V proteins of henipaviruses sequester STAT1 and prevent their activation [24, 25]; and measles virus V protein blocks IFN-induced STAT1/2 nuclear translocation by an unknown mechanism [26].

Nipah virus (NiV) is a newly emerging and a highly pathogenic zoonotic paramyxovirus that causes fatal diseases in humans [27, 28]. NiV P, V and W proteins have been demonstrated to block IFN-I signaling and to bind STAT1 [24, 29]. W protein of NiV inhibits the host IFN response by sequestering STAT1 in the nucleus [30–32] and by blocking virus and TLR3-dependent ISG induction and TBK1/IKKε-mediated IRF3 activation [33]. However, although NiV is known to inhibit IFN responses during infection, studies have shown that mutations in NiV-P or its gene products fail to abolish inhibition of STAT activation and recombinant viruses harboring these mutations are not attenuated in IFN competent cells [34–37]. Therefore, additional NiV proteins may also be able to inhibit RIG-I-mediated IFN production.

Here, we show that the matrix protein of NiV (NiV-M) has a role in IFN antagonism and that a conserved lysine residue (K258) in the bipartite nuclear localization signal (NLS) of NiV-M is essential for this activity. Interestingly, this residue has been implicated in the ubiquitin-regulated nucleo-cytoplasmic shuttling of NiV-M in the early stages of Ni-V infection [38]. Mutation of the conserved lysine to either alanine (K258A) or arginine caused defects in nuclear import or export respectively; both mutants had decreased levels of ubiquitination and budding defects [38, 39]. We show that K258 is essential for NiV-M to counteract IFN-I mediated responses, as a K258A mutant failed to inhibit IFN-I production and the induction of ISGs via disrupting IKKε activation. Mechanistically, this is achieved by NiV-M-induced degradation of both endogenous and overexpressed TRIM6, which we had previously reported to be involved in activating IKKε via endogenous K48-linked unanchored polyubiquitin chains [13].

## Results

### Nipah virus matrix protein inhibits IFNβ induction at the level of the TBK1/IKKε kinases

**Fig 1:**
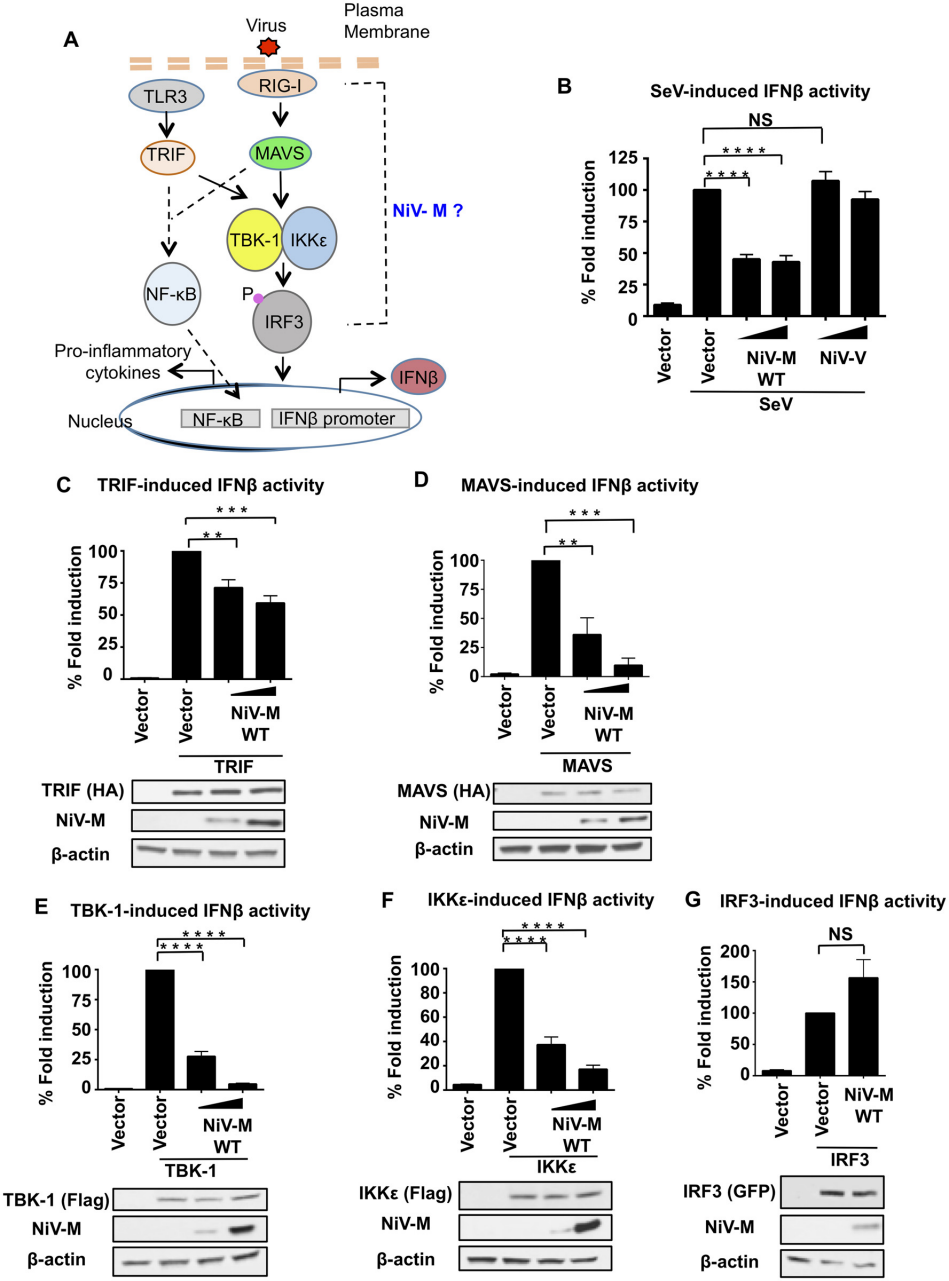
Nipah virus matrix protein inhibits IFNβ induction at the level of the TBK1/IKKε kinases. **A)** Schematics of the signaling pathways investigated. (B-G) HEK293T cells were transfected with a Luciferase reporter plasmid under the control of the IFNβ promoter and a Renilla control plasmid, in the presence or absence of NiV-M and stimulated with SeV (B), or transfected with stimulating plasmids TRIF (C), MAVS (D), TBK-1 (E), IKKε (F) or IRF3 (G), followed by luciferase assay. Data were normalized first by none stimulated sample to obtain fold induction, and then stimulated samples were set to 100% to obtain percentage of fold induction. Data are from three independent experiments; each one in triplicate, and depicted is the mean ± SD (n = 9). *p < 0.05; **p < 0.01; ***p < 0.001, ****p < 0.0001, by Student’s t test. Immunoblots using lysates from transfected samples are shown as controls.

Nipah virus encodes for four viral proteins (P, C, V, W) with IFN antagonist functions when used in overexpression studies [34, 40]. Although it is now clear that NiV is able to inhibit IFN responses during virus infection, recombinant NiV with mutations in the P, C, V or W genes are not significantly attenuated in IFN-competent cells [34–37], suggesting that NiV encodes for additional viral proteins with ability to antagonize the IFN system. The NiV matrix protein, which is required for virus budding and assembly, has been shown to traffic through different cellular compartments before being targeted to the cellular membrane for virus assembly [38, 39], raising the question as whether it may have non-structural functions. We hypothesized that NiV-M is a viral product with novel innate immune antagonist functions. To test this hypothesis, the ability of NiV-M to inhibit different components of the type-I IFN pathway was investigated (Fig 1A). Exogenous NiV-M expression reduced Sendai virus (SeV)-induced IFNβ promoter activation in a reporter assay (Fig 1B). In contrast, NiV-V protein, which is known to inhibit IFN signaling but not IFN production, did not affect IFNβ induction (Fig 1B). In line with these results and a role of IFNβ in inducing antiviral responses, SeV RNA replication levels were increased in M expressing cells (S1 Fig). NiV-M also blocked IFNβ induction mediated by TRIF and MAVS (Fig 1C and 1D), albeit with different potencies. TRIF and MAVS act as adaptor proteins for the TLR3 and RIG-I pathways respectively (see diagram in Fig 1A). Since MAVS and TRIF dependent signaling pathways converge downstream at the level of the TBK1/IKKε kinases, these results suggest that NiV-M may target shared signaling factors downstream of these adaptor proteins. Consistent with this hypothesis, NiV-M also inhibited TBK-1- and IKKε-dependent IFN induction in a dose-dependent manner (Fig 1E and 1F). In contrast, NiV-M did not inhibit the IRF3-induced IFNβ reporter activation (Fig 1G), suggesting that NiV-M blocks IFN induction by acting at the level of the TBK-1/IKKε kinases.

**Fig 2:**
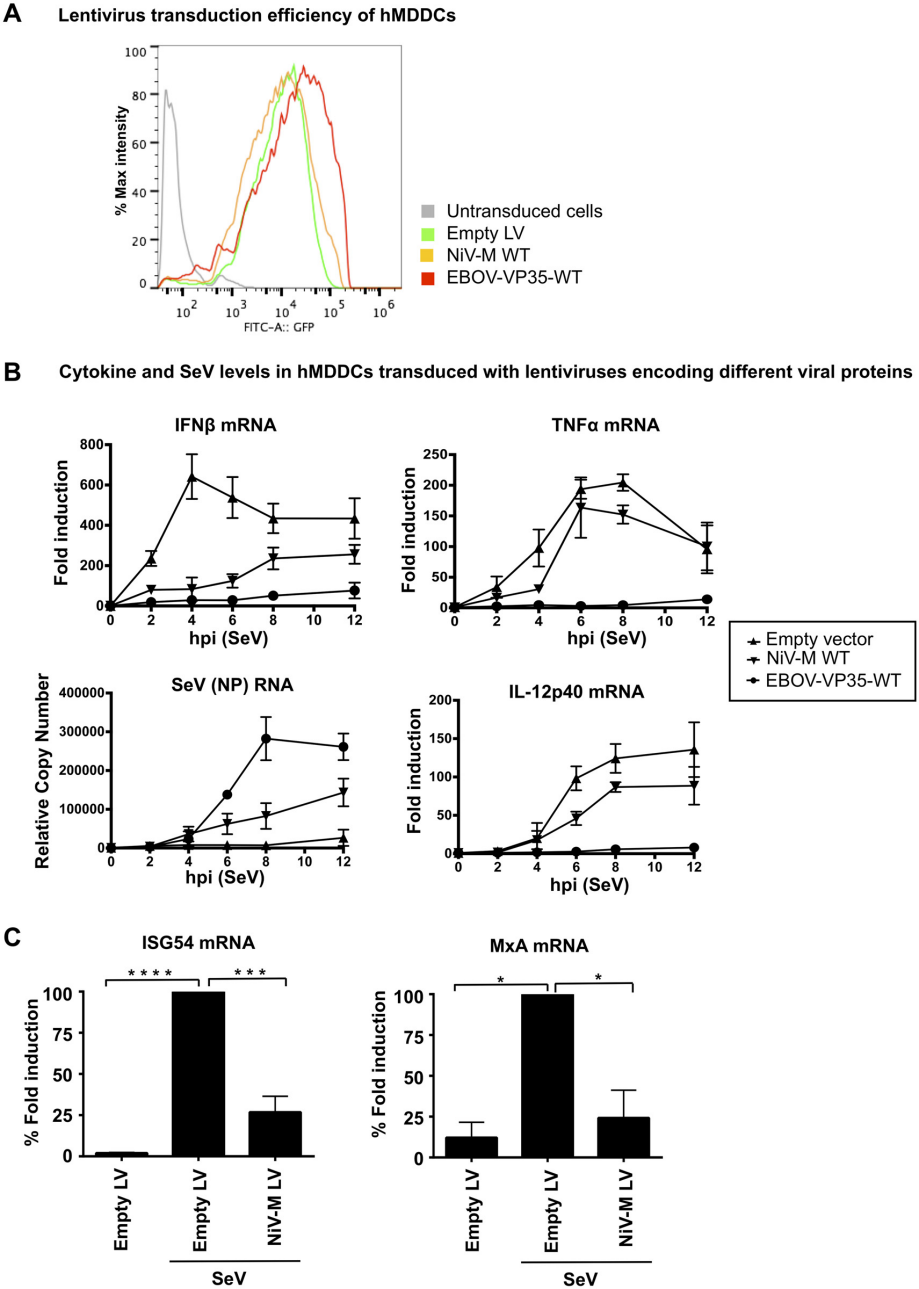
NiV-M protein inhibits IFNβ but not the pro-inflammatory cytokines TNFα and IL-12p40 in primary human MDDC. **A)** Human MDDCs were either mock transduced, transduced with Vpx-VLPs only, or cotransduced with lentiviral (LV) vectors expressing NiV-M or Ebola virus VP35 and Vpx-VLPs. hMDDCs were harvested at day 5 post-transduction for GFP analysis by flow cytometry. B) hMDDCs where then stimulated with SeV and total RNA was isolated and analyzed by qRT-PCR to quantify IFNβ, SeV NP RNA, TNFα, and IL-12p40 levels or C) ISG54 and MxA. RPS11 was used as housekeeping gene and mRNA levels are shown as fold induction over mock treated samples. Error bars indicate standard deviations. Samples were collected from three independent donors. *p < 0.05; **p < 0.01; ***p < 0.001, ****p < 0.0001, by Student’s t test.

To further validate these results and ensure that NiV-M has a biological function in relevant primary innate immune cells, experiments were performed in primary human monocyte-derived dendritic cells (hMDDC), which are targets of NiV infection [41]. To this end, lentiviruses encoding the NiV-M protein and the well-characterized IFN antagonist VP35 protein from Ebola virus [42, 43] were generated as previously described [42, 44], and their ability to inhibit IFN responses was examined. Monocytes from healthy donors were cultured with GMCSF and IL-4 for 5 days followed by lentiviral transduction [42, 44]. The efficiency of lentiviral transduction was monitored by flow cytometry (Fig 2A). hMDDC were then challenged with the Cantell strain of SeV, which activates the RIG-I pathway via its known production of viral-defective interfering particles (DI), leading to induction of IFNβ and pro-inflammatory cytokines. IFNβ mRNA expression peaked at 4 hr post-infection (p.i.) and was induced about 600-fold (Fig 2B, left panel). NiV-M-expressing cells potently inhibited IFNβ induction as compared to empty lentiviral control vector (Fig 2B, top left panel), whereas SeV RNA replication was increased (Fig 2B, bottom left panel). Consistent with the previously reported role of VP35 on RIG-I function [45], induction of the pro-inflammatory cytokines TNFα and IL-12p40, were reduced in VP35 expressing cells. In contrast, NiV-M expression had only minor effects on TNFα and IL-12p40 expression levels as compared to the control lentiviral vector (Fig 2B, top and bottom right panels). Since these pro-inflammatory cytokines require activation of the NF-kB transcription factor and these signaling pathways diverge downstream of MAVS/TRIF (see diagram in Fig 1A), these results are consistent with our reporter assays and support a role of NiV-M downstream of the adaptor proteins and towards the TBK-1/IKKε signaling axis. Furthermore, consistent with reduced IFNβ induction in NiV-M-expressing cells, the induction of IFN-stimulated genes ISG54 and MxA was also attenuated as compared to control cells (Fig 2C).

Taken together these data indicate that NiV-M specifically acts as a potent antagonist of IFNβ and antiviral responses but does not play a significant role in inhibition of pro-inflammatory cytokines in cell lines and relevant primary innate immune cells.

### NiV matrix protein inhibits IKKε activation

**Fig 3:**
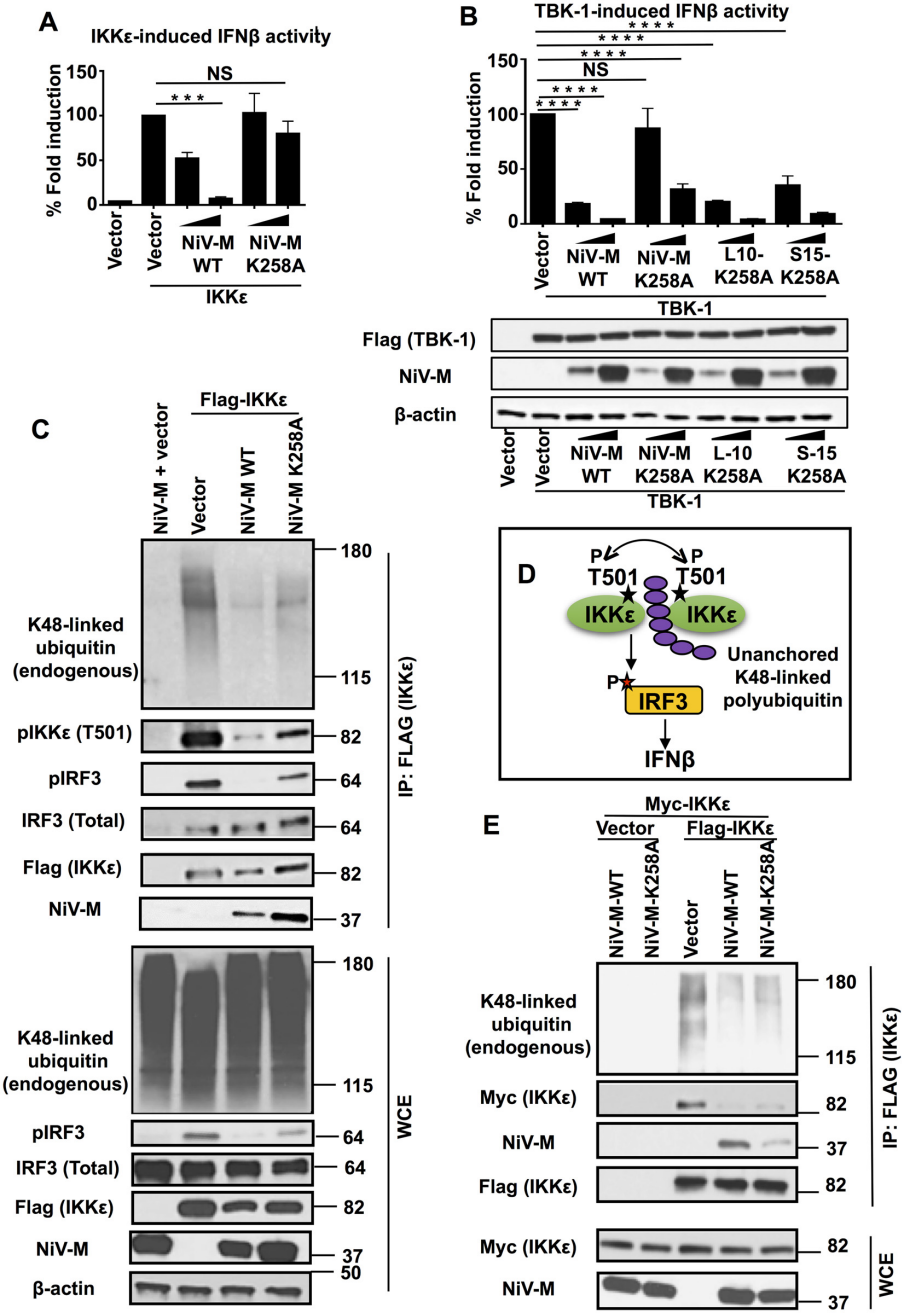
Lysine K258A on NiV matrix protein is important for inhibition of unanchored K48-linked polyubiquitin chains that associate with IKKε and required for IKKε activation. (A-B) NiV-M requires membrane targeting for inhibition of IKKε/TBK-1 mediated IFNβ induction. HEK293T cells were transfected with IFNβ luciferase reporter in the presence of increasing concentrations of NiV-M WT or a K258A mutant and stimulating plasmid IKKε (A) or TBK-1 and the membrane targeting fusion proteins of M-K258A (S10-K258A or S15-K258A) (B). C) NiV-M-WT inhibits association of unanchored K48-linked polyubiquitin chains with IKKε leading to reduced IKKε-T501 phosphorylation and reduced IRF3 phosphorylation. HEK293T cells were transfected with NiV-M or NiV-M-K258A mutant in the presence or absence of Flag-IKKε. Cells were harvested and whole cell extracts (WCE) were used for IKKε immunoprecipitation using anti-Flag beads. A representative of at least 3 independent experiments is shown. Note that the WCE blot for K48-linked ubiquitin represents the levels of the total cellular pool of K48-linked ubiquitin (covalently ubiquitinated proteins and unanchored), which do not change in the presence of NiV-M. Only K48-linked ubiquitin that specifically associates with IKKε is reduced in the presence of NiV-M (shown in the IP panel). D) Schematics of IKKε activation by unanchored K48-linked polyubiquitin chains as described [13]. E) NiV-M-WT but not K258A inhibits oligomerization of IKKε. HEK293T cells were transfected with Flag-IKKε and Myc-IKKε in the presence of NiV-M, NiV-M-K258A or empty vector control. Oligomerization of IKKε was assessed by the ability of IKKε to interact with itself by immunoprecipitation. IKKε was immunoprecipitated from WCE with anti-Flag beads followed by immunoblot with Myc antibody. Representatives of at least 2 independent experiments are shown. *p < 0.05; **p < 0.01; ***p < 0.001, ****p < 0.0001, by Student’s t test.

NiV-M travels to the cell membrane where it is required for viral assembly and budding during viral replication [38]. However, the NiV-M protein traffics from the cytoplasm to the nucleus before reaching the cell membrane. Translocation to the nucleus requires a conserved lysine residue (K258) in the bipartite nuclear localization signal (NLSbp) of the NiV-M protein, and a lysine to alanine mutant (K258A) results in retention of NiV-M in the non-membrane cytoplasmic fraction [38]. To further understand the mechanism by which NiV-M inhibits IKKε activity, we tested whether NiV-M-K258A mutant has the ability to inhibit IFN induction. IKKε-mediated IFN induction was attenuated approximately 50% in the presence of low concentrations of NiV-M-WT. In contrast, NiV-M-K258A did not inhibit IFN induction in these conditions (Fig 3A). Although high concentrations of M-K258A marginally inhibited IFN induction, these effects are significantly attenuated as compared to NiV-M-WT (Fig 3A). Similar effects were observed when cells were activated with TBK-1; however, TBK-1 appeared to be more sensitive to the inhibitory effects of M as compared to IKKε, and higher concentrations of M-K258A were able to inhibit IFN induction (Fig 3B).

Since the K258A mutant of NiV-M is retained in the non-membrane cytoplasm fraction [38], it could be that its loss of IFN antagonist function is due to failure to reach the cytoplasmic/membrane compartments where the IFN-I signaling components localize (for example, MAVS localizes to the mitochondrial membrane; [46]). To test this possibility, we utilized two different M-K258A fusion proteins that contain membrane-targeting signals (L10-K258A and S15-K258A) and have been previously shown to rescue M-K258A trafficking to the cytoplasmic membranous fractions [38]. Strikingly, ectopic expression of either L10-K258A or S15-K258A rescued the loss of inhibitory effects of NiV-M-K258A on TBK-1-induced IFNβ expression, especially at low dose of M expressing vectors (Fig 3B). Of note, all NiV-M proteins were expressed in similar levels and TBK-1 levels were not affected in the presence of NiV-M-WT or mutant M proteins (Fig 3B, bottom panel). These results suggest that NiV-M requires trafficking to cytoplasmic/membrane fractions for antagonism of IFN-I responses.

It appeared that NiV-M-K258A had stronger inhibitory effects on TBK-1 as compared to IKKε (compare NiV-M-WT to NiV-M-K258A in Fig 3A and 3B). Therefore, the K258A mutant provides a better tool to study the mechanism by which NiV-M inhibits IFN induction mediated by IKKε. Furthermore, we recently elucidated the detailed molecular mechanism by which IKKε is activated [13]. Therefore, we focused our study on the IKKε kinase.

We have shown that NiV-M inhibits IKKε but not IRF3-mediated IFN induction (Fig 1F and 1G), suggesting that M targets the IKKε kinase. In support of this, co-immunoprecipitation (coIP) studies demonstrated that both NiV-M-WT and the K258A mutant efficiently interacted with IKKε when coexpressed in HEK293T cells (Fig 3C). Although NiV-M did not disrupt binding of IKKε to IRF3, IKKε-mediated phosphorylation of IRF3 was reduced in the presence of WT-NiV-M, while only minor effects were observed in the presence of NiV-M-K258A (Fig 3C, pIRF3 row), suggesting that NiV-M-WT inhibits IKKε activity and that the K258 residue is required for this effect. Consistent with this, autophosphorylation of IKKε on T501 (a marker of IKKε activation) was reduced in the presence of NiV-M-WT as compared to NiV-M-K258A or the empty vector control (Fig 3C, quantifications shown in S2 Fig).

We recently reported that IKKε activity is regulated by unanchored K48-linked polyubiquitin chains, which are not covalently attached to any protein [13]. These polyubiquitin chains interact with IKKε and promote its oligomerization and autophosphorylation on IKKε-T501 (see diagram in Fig 3D; [13]). NiV-M-WT reduced association of IKKε with K48-linked unanchored polyubiquitin chains to a greater degree than M-K258A (Fig 3C and 3E). In line with these results, IKKε oligomerization was also reduced in the presence of NiV-M-WT as compared to empty vector or the NiV-M-K258A mutant (Fig 3E). It is important to note that expression of NiV-M does not reduce the total cellular pool of K48-linked polyubiquitin chains, which include covalently ubiquitinated proteins and unanchored polyubiquitin chains (see Fig 3C, panel K48-linked ubiquitin, WCE). Together these results indicate that NiV-M-WT blocks activation of IKKε by suppressing its specific association with unanchored K48-linked polyubiquitin chains, which in turn inhibits downstream IKKε oligomerization and IRF3 phosphorylation.

**Fig 4:**
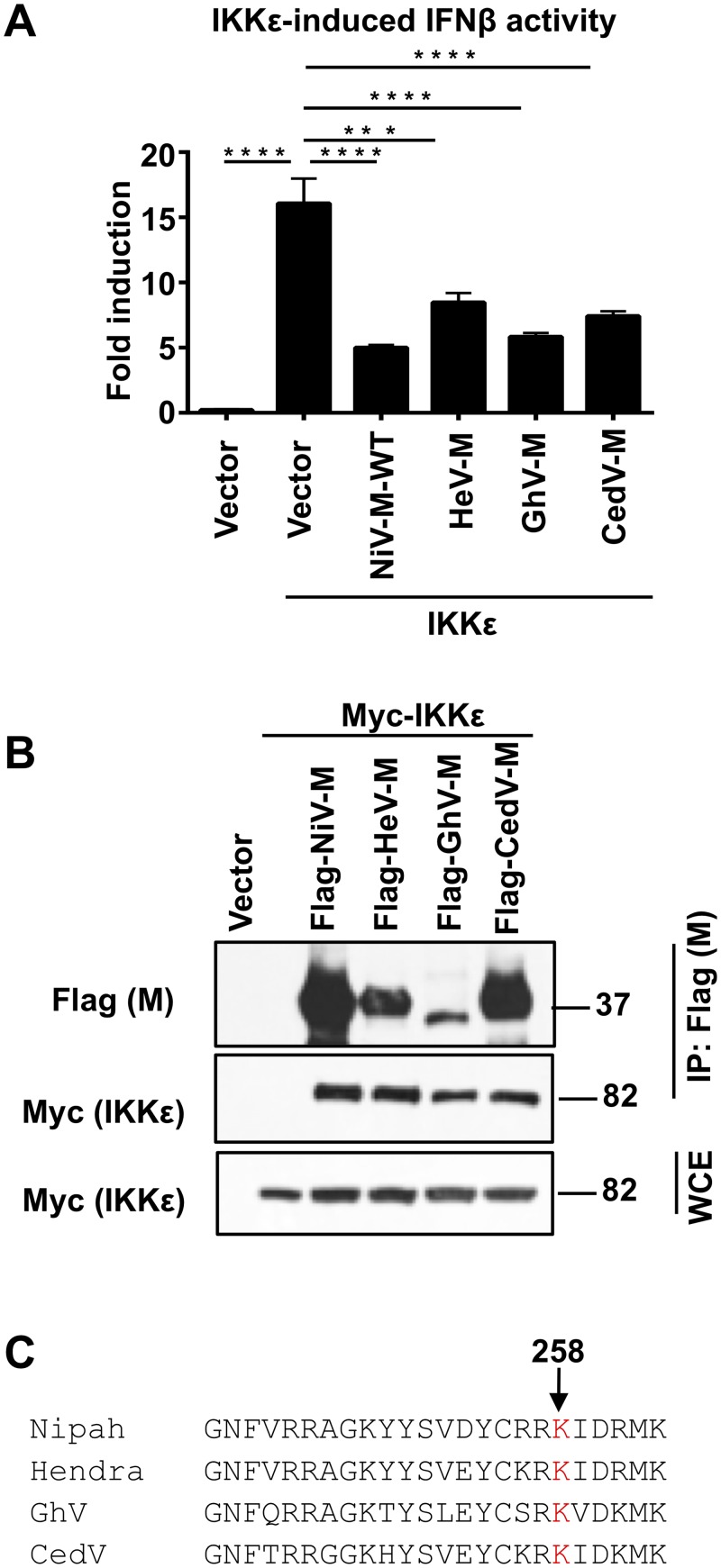
Matrix proteins from henipaviruses with conserved Lysine K258A interact with IKKε and inhibit IKKε-mediated IFNβ induction. A) Matrix proteins from henipaviruses inhibit IKKε-mediated IFNβ induction. HEK293T cells were transfected with IFNβ luciferase reporter and a Renilla control plasmid, in the presence or absence of NiV-M, HeV-M, GhV-M or CedV-M and IKKε, followed by luciferase assay. Data were normalized by none stimulated sample to obtain fold induction. Depicted is the mean ± SD (n = 3). B) Matrix proteins of henipaviruses interact with IKKε. HEK293T cells were transfected with Flag-NiV-M, Flag-HeV-M, Flag-GhV-M or Flag-CedV-M in the presence or absence of Myc-IKKε. Cells were harvested and whole cell extracts (WCE) were used for M immunoprecipitation using anti-Flag beads. C) Protein sequence alignment of the region mapping to the nuclear localization signal (NLS) of Nipah virus (NiV), Hendra Virus (HeV), Ghana virus (GhV) and Cedar virus (CedV). *p < 0.05; **p < 0.01; ***p < 0.001, ****p < 0.0001, by Student’s t test.

To test whether other henipavirus matrix proteins also have IFN antagonist functions, we examined the ability of the matrix proteins of Hendra virus (HeV), Ghana virus (GhV) and Cedar virus (CedV) to inhibit IFNβ induction in our reporter assays and their ability to bind IKKε in coIP assays. GhV is a novel African bat henipavirus that is divergent from NiV and HeV (~ 60% sequence identity in the M gene compared to ~90% identity between NiV-M and HeV-M) [47, 48]. CedV, which is the most recent member of the genus *Henipavirus*, has been reported to be non-pathogenic in ferrets and guinea pigs and has reduced ability to inhibit IFN signaling, presumably by absence of its P gene products [49, 50]. HeV-M, GhV-M and CedV-M significantly inhibited IKKε-induced IFN promoter activation (Fig 4A). Furthermore, the matrix proteins of these three henipaviruses also interacted with IKKε (Fig 4B). These results demonstrate that the matrix proteins of henipaviruses have IFN antagonist functions. The conservation of NLSbp in the henipavirus matrix proteins suggests that the NLS region is potentially important for this function (Fig 4C) [38].

### NiV matrix protein interacts with the E3-ubiquitin ligase TRIM6 and inhibits TRIM6-mediated IFN responses

We have shown that NiV-M expression results in reduced levels of the unanchored K48-linked polyubiquitin chains that specifically associate with IKKε (Fig 3C). This effect could be due to either interference of IKKε binding to unanchored polyubiquitin chains or by inhibition of the enzyme that synthesizes these polyubiquitin chains.

**Fig 5:**
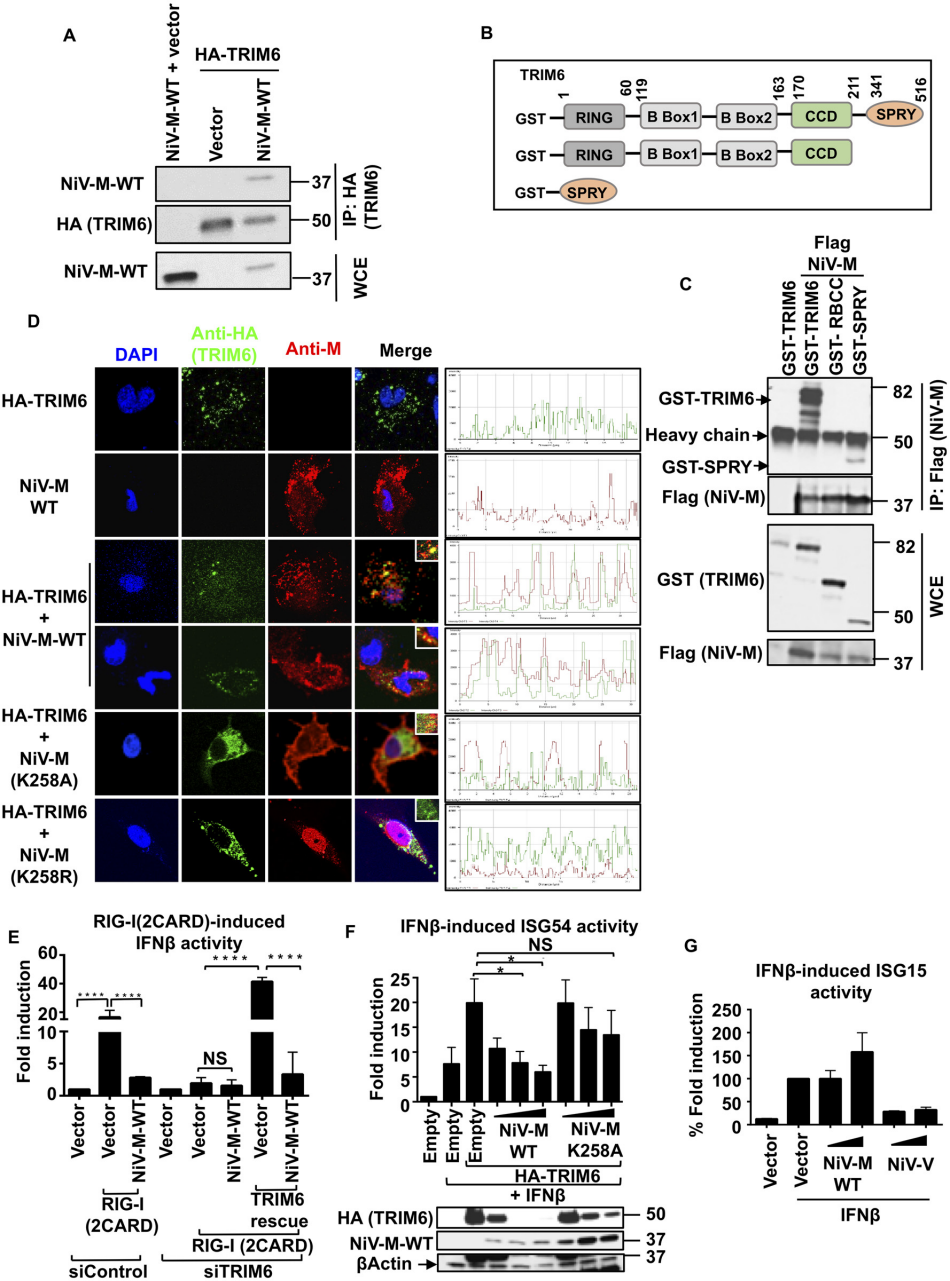
NiV-M interacts with TRIM6 and inhibits TRIM6-medited IFNβ induction and signaling. A) NiV-M interacts with TRIM6. HEK293T cells were transfected with NiV-M, empty vector or HA-TRIM6. Cells were harvested and whole cell extracts (WCE) were used for TRIM6 immunoprecipitation using anti-HA-beads. (B-C) the C-terminal SPRY domain of TRIM6 interacts with NiV-M. B) TRIM6 deletion mutants used for coIP (GST-tagged). C) HEK293T cells were transfected with the GST-TRIM6 deletion mutants indicated together with NiV-M. Cells were harvested and WCE were used for TRIM6 immunoprecipitation using GST-beads. D) NiV-M co-localizes with TRIM6 in cytoplasmic bodies. HeLa cells were transfected with HA-TRIM6 and NiV-M-WT or NiV-M mutants K258A or K258R. Cells were fixed and stained with the indicated antibodies followed by confocal microscopy. Colocalization profiles are shown on the right. E) NiV-M inhibits the RIG-I induced IFNβ by targeting TRIM6. HEK293T were transfected with a non-targeting siRNA control (siControl) or aTRIM6-targeting siRNA (siTRIM6). After 24 hours, cells were transfected with IFNβ luciferase reporter and a Renilla control plasmid, in the presence or absence of NiV-M, or reconstitution with HA-TRIM6, followed by luciferase assay. Data were normalized by none stimulated sample to obtain fold induction. Depicted is the mean ± SD (n = 3). (F-G) NiV-M inhibits TRIM6-mediated IFNβ-induced ISG54 but not ISG15 reporter activity. HEK293T cells were transfected with ISG54-ISRE luciferase reporter (F) or ISG15 luciferase reporter (G) and a Renilla control plasmid and HA-TRIM6 in the presence of increasing concentrations of NiV-M-WT or NiV-MK258A, followed by luciferase assay. Data were normalized by none stimulated sample to obtain fold induction. Depicted is the mean ± SD (n = 3). Representatives of at least 2 independent experiments are shown. *p < 0.05; **p < 0.01; ***p < 0.001, ****p < 0.0001, by Student’s t test.

We recently demonstrated that TRIM6, a member of the TRIM E3-ubiquitin ligase family of proteins, catalyzes the synthesis of unanchored K48-linked polyubiquitin chains that specifically associate with IKKε promoting its oligomerization and activation for downstream signaling [13]. We hypothesized that NiV-M blocks IKKε activation by inhibiting the TRIM6-mediated synthesis of unanchored polyubiquitin chains. To this end, we first tested whether NiV-M interacts with TRIM6 in coIP assays. In line with our hypothesis, NiV-M efficiently interacted with TRIM6 (Fig 5A). To map the region of TRIM6 binding to NiV-M, we used deletion mutants of TRIM6 expressing the N-terminal RING (R), B box and coiled-coil (CC) domains or the C-terminal SPRY domain (diagram in Fig 5B) and tested interaction with NiV-M. The C-terminal SPRY domain of TRIM6 specifically interacted with NiV-M-WT, whereas a mutant lacking only the SPRY domain lost the ability to interact with NiV-M (Fig 5C), indicating that this interaction required the C-terminal SPRY domain of TRIM6. Since IKKε also interacts with the SPRY domain of TRIM6 [13], we asked whether NiV-M competes with IKKε for TRIM6 binding. In agreement with this possibility, coIP studies showed that the interaction between TRIM6 and IKKε is decreased in the presence of both NiV-M-WT and NiV-M-K258A (S3 Fig), suggesting that inhibition of IKKε activity can be explained in part by interference of TRIM6-IKKε binding by NiV-M, and that this interference does not depend on the NiV-M K258 amino acid.

To further confirm binding of TRIM6 with NiV-M, we performed co-localization studies by confocal microscopy (Fig 5D). As previously reported [13, 51], TRIM6 localizes in punctate cytoplasmic bodies. In contrast NiV-M-WT showed membrane localization as well as some nuclear and cytoplasmic localization (Fig 5D), as previously shown [38]. However, upon NiV-M and TRIM6 coexpression, a fraction of NiV-M colocalized with TRIM6 in cytoplasmic bodies. The NiV-M K258A mutant, which does not translocate to the nucleus, also appeared to colocalize with TRIM6 (Fig 5D). Importantly, a K258R mutation on NiV-M, which translocates to the nucleus but does not exit the nucleus [38], did not colocalize with TRIM6 (Fig 5D). It is important to note that cells coexpressing NiV-M-WT and TRIM6 appeared to have lower levels of TRIM6 and in many cases it was difficult to observe the characteristic punctate localization of TRIM6 (Fig 5D, see next section for more details).

To test whether NiV-M binding to TRIM6 has functional relevance, we examined the ability of NiV-M to inhibit IFNβ promoter activation by the constitutively active 2CARD domain of RIG-I [RIG-I(2CARD)] [3, 4] in TRIM6 knockdown cells. As expected, NiV-M inhibited RIG-I(2CARD)-induced IFNβ promoter activation in non-targeting siRNA control cells (Fig 5E). TRIM6-targeting siRNA cells showed a markedly reduced IFNβ promoter activity upon RIG-I(2CARD) transfection compared to control cells, as we previously reported [13]; however there was a minor but still detectable IFNβ induction by RIG-I(2CARD) stimulation. This residual IFNβ induction was not significantly affected by exogenous NiV-M expression (Fig 5E). Reconstitution of TRIM6 rescued the RIG-I(2CARD)-induced IFNβ reporter activity in TRIM6 knockdown cells and this induction was almost completely blocked in the presence of NiV-M (Fig 5E), demonstrating that M inhibits IFN induction by a TRIM6-dependent mechanism.

In addition to its role in phosphorylation of IRF3, the IKKε kinase has been shown to play a non-redundant role in the type-I IFN signaling pathway and is required for the expression of a subset of IKKε-dependent ISGs [14]. As we previously reported [13], ectopic expression of TRIM6 enhances the IFNβ-induced ISG54 reporter activity (ISG54 is an IKKε-dependent ISG) (Fig 5F). In support for a role for NiV-M in inhibition of the TRIM6-IKKε axis in the IFN signaling pathway, NiV-M-WT but not the M-K258A was able to inhibit TRIM6-mediated IFNβ-induced ISG54 reporter activity in a dose-dependent manner (Fig 5F). Importantly, the levels of TRIM6 protein were also reduced by NiV-M-WT in a dose-dependent manner, whereas TRIM6 protein levels were less affected in the presence of NiV-M-K258A (Fig 5F, immunoblot). These results suggest that NiV-M inhibits IFN responses by reducing TRIM6 protein expression. NiV-M did not have a general effect on inhibition of the IFN signaling pathway because, in contrast to the effects observed on the ISG54 reporter, NiV-M did not inhibit the IFNβ-induced ISG15 reporter activity, as ISG15 is not an IKKε-dependent ISG (Fig 5G, and [13, 14]. The NiV-V protein, which is known to target STAT1 for antagonism of IFN signaling [24, 25], reduced ISG15 reporter activity and was used as a positive control for this experiment (Fig 5G).

Taken together these data indicate that NiV-M acts as an antagonist of both the IFN production and IFN signaling pathways by targeting the E3-ubiquitin ligase TRIM6 and consequently blocking the activation of IKKε.

### NiV-M targets TRIM6 for degradation

Our results show that TRIM6 and NiV-M interact in coIP assays and colocalize in cytoplasmic bodies (Fig 5A–5D). While performing these experiments we noticed a reduction of TRIM6 protein levels in the presence of NiV-M-WT (see Fig 5A, 5D and 5F), suggesting that TRIM6 may be targeted for degradation. Evidence included a very low number of cells in which TRIM6 and NiV-M-WT were detected together in the colocalization studies. In the rare instance in which NiV-M-WT and TRIM6 were detected in the same cell, TRIM6 levels appeared lower and did not exhibit its characteristic punctate localization as compared to the TRIM6 dots observed when expressed alone (Fig 5D). The effect of NiV-M on TRIM6 expression levels was confirmed in biochemical assays in which expression of NiV-M-WT clearly reduced the levels of ectopically expressed TRIM6 in a dose-dependent manner. This effect on TRIM6 levels was significantly attenuated in the presence of the NiV-M-K258A mutant (see immunoblot in Fig 5F and intensity of the TRIM6 dots in 5D, M-K258A).

**Fig 6:**
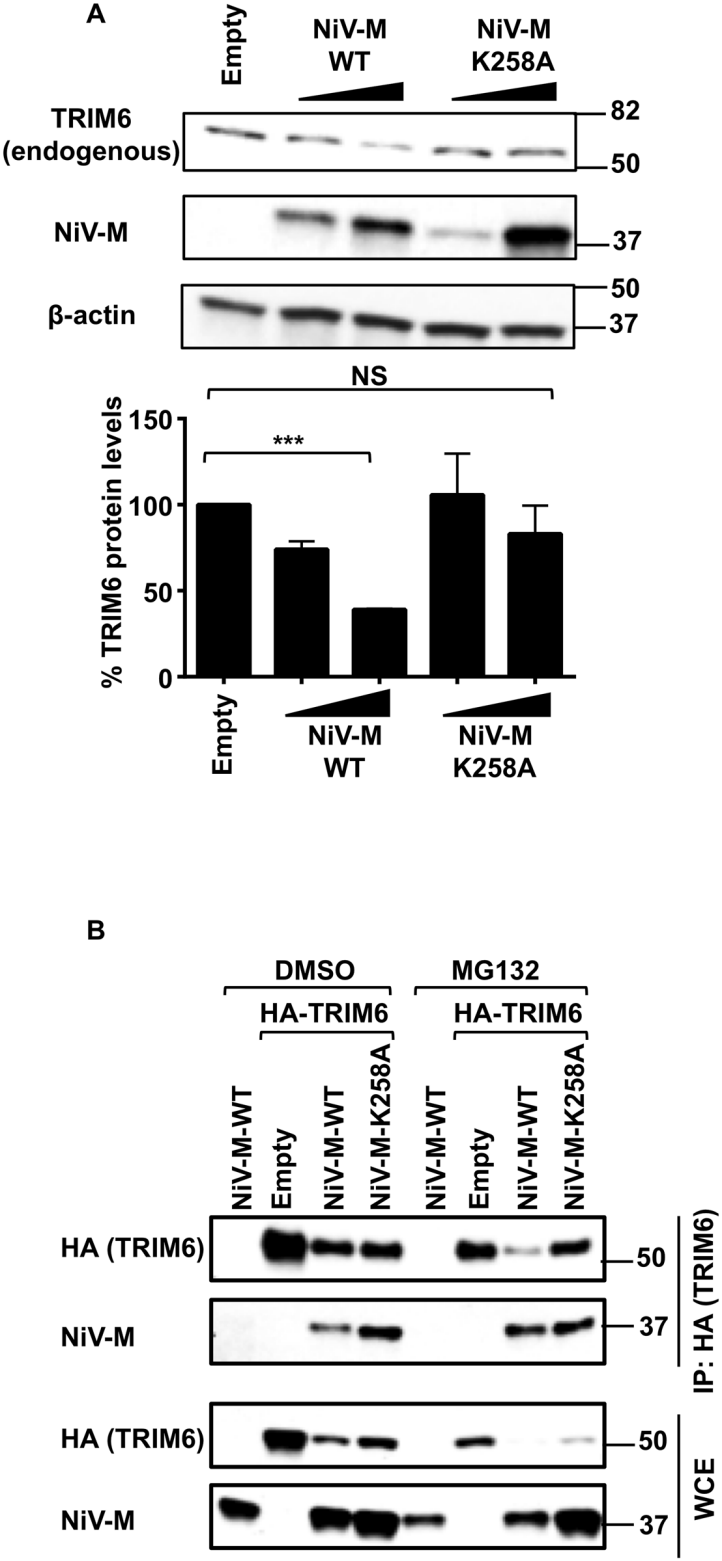
NiV-M targets TRIM6 for degradation. A) HEK293T cells were transfected with increasing concentrations of NiV-M WT or a K258A mutant and the levels of endogenous TRIM6 were determined by immunoblot (representative of 3 experiments). For quantification, the levels of TRIM6 were normalized by actin, which were determined using ImageJ software. Data is combination of 3 independent experiments. *p < 0.05; **p < 0.01; ***p < 0.001, ****p < 0.0001, by Student’s t test. B) HEK293T cells were transfected with NiV-M-WT or NiV-M-K258A, empty vector and HA-TRIM6. Twenty-four hours post-transfection cells were treated with DMSO or the proteasome inhibitor MG132 for 4 hrs. Cells were harvested and whole cell extracts (WCE) were used for TRIM6 immunoprecipitation using anti-HA-beads.

To rule out possible artifacts of TRIM6 overexpression, the levels of endogenous TRIM6 were quantified in the presence of NiV-M-WT or NiV-M-K258A. In agreement with our observations, endogenous TRIM6 protein levels were also reduced in NiV-M-WT expressing cells, whereas the K258A mutant did not affect TRIM6 protein levels (Fig 6A). Similar results were observed in coIP assays, and proteasome inhibition with MG132 did not recover TRIM6 protein (Fig 6B), suggesting that NiV-M promotes TRIM6 degradation by a proteasome-independent mechanism.

To validate these observations in the context of live Nipah virus infections, we generated recombinant NiV (rNiV) lacking the M protein (rNiV-ΔM) and compared the levels of endogenous TRIM6 during rNiV-WT and rNiV-ΔM infections. To facilitate detection of infected cells by immunofluorescence, both WT and ΔM rNiVs were engineered to express GFP. Since the M protein is required for virus budding and assembly, rNiV-ΔM was initially rescued in HEK293 cells stably expressing the M protein *in trans* [38, 39]. However, rNiV-ΔM could be passaged on Vero cells in the absence of exogenous M, albeit with much delayed replication kinetics compared to WT rNiV-GFP (S4A Fig), in agreement with a recent study [52]. In rNiV-WT infected cell lysates, both nucleocapsid and M proteins could be detected as early as 24 h.p.i, whereas no M protein could be detected in rNiV-ΔM infected cell lysates at any of the time points tested, even though by 48 h.p.i, virus replication was evident by the presence of nucleocapsid protein (S4B Fig). These results also confirm that our rNiV-ΔM virus is truly M-deficient.

**Fig 7:**
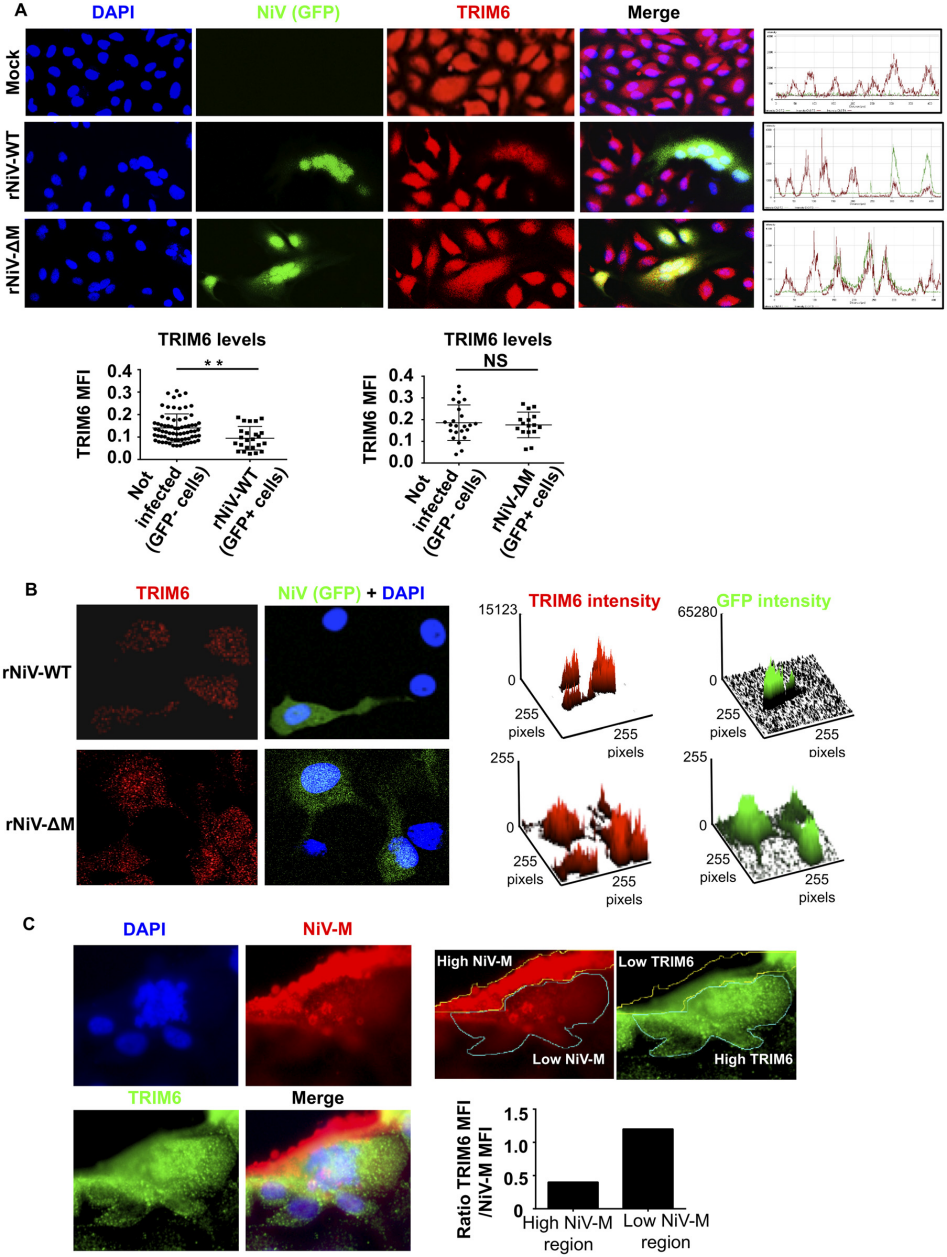
NiV-M targets TRIM6 for degradation during NiV infection. A) HeLa cells were infected with NiV-WT or NiV lacking the matrix protein (NiV-ΔM), expressing GFP, at MOI of 0.1. Forty-eight hours p.i. cells were fixed and stained with a TRIM6 antibody, followed by secondary anti-rabbit-555 (red) and immunofluorescence. Fluorescence intensity profiles are shown on the right. Mean Fluorescence Intensity (MFI) was quantified from three different images using ImageJ and graphs are shown below. TRIM6 MFI values were obtained from individual GFP+ or GFP- cells. For this quantification, individual cells were selected using the freehand selection tool in ImageJ software following the borders of each individual cell by following TRIM6 staining. Over 50 cells were selected for quantification in ImageJ software. B) Confocal microscopy of endogenous TRIM6 (red dots) in NiV-M-WT and NiV-ΔM infected cells. NiV-M-WT, GFP+ cells, have reduced levels of TRIM6 (MFI) and number of TRIM6 dots as compared to non-infected GFP- cells (quantification on the right). NiV-M-ΔM GFP+ infected cells, do not show significant differences in TRIM6 levels (MFI) or number of TRIM6 dots as compared to non-infected GFP- cells. A surface plot is shown, obtained using ImageJ based on pixels from the confocal images. C) NiV-M infected samples were stained with anti-M antibody (red) and anti-TRIM6 (for presentation TRIM6 is depicted in green). The region of high and low M expression was selected manually using the freehand tool in ImageJ. The region of high M expression correlates with very low TRIM6 staining as well as no visible TRIM6 dots. TRIM6 dots can clearly be seen in the cellular regions with low or no M expression. For quantification, the TRIM6 fluorescence intensity values in the low and high M areas where normalized by the MFI levels of M. *p < 0.05; **p < 0.01; ***p < 0.001, ****p < 0.0001, by Student’s t test.

Next, we compared the effects of these rNiVs on endogenous TRIM6 expression. Consistent with a role of M in TRIM6 degradation, cells infected with rNiV-WT (GFP+ cells, Fig 7A) expressed lower levels of TRIM6 protein as compared to uninfected cells (GFP- cells, Fig 7A). In contrast, cells infected with rNiV-ΔM showed similar levels of TRIM6 protein expression as compared to uninfected cells (Fig 7A). Furthermore, the number and intensity of TRIM6 cytoplasmic dots also appeared to be reduced as compared to non-infected cells, while no significant differences were observed in rNiV-ΔM infected versus non-infected cells (Fig 7B). In line with these results, TRIM6 expression and dot formation was excluded from areas with high levels of matrix protein in rNiV-WT infected cells (Fig 7C). These data demonstrate that NiV-M reduces the protein levels of TRIM6 during virus infection.

**Fig 8:**
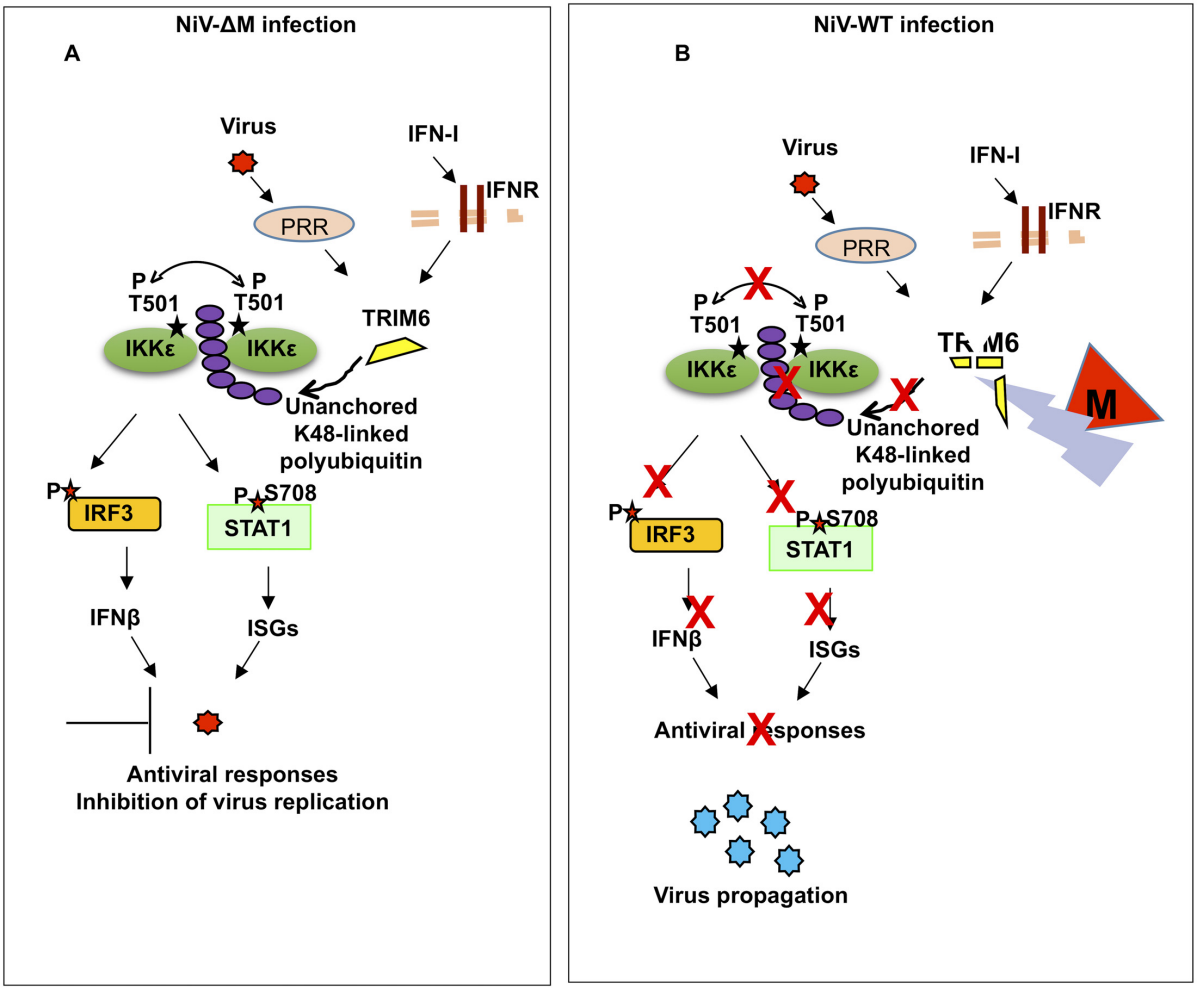
Proposed model of NiV-M inhibition of IFN responses. A) Upon virus recognition in Nipah virus infected cells lacking the matrix protein, PRR signaling promotes the synthesis of unanchored K48-linked polyubiquitin chains by the E3-ubiquitin ligase TRIM6. These polyubiquitin chains interact with IKKε and induce its oligomerization and T501 autophosphorylation. Consequently, activated IKKε phosphorylates IRF3 resulting in IFNβ induction and establishment of an antiviral response. IFNβ signaling through its receptor can also lead to TRIM6 and IKKε activation for induction of ISGs. B) The presence of NiV-M-WT promotes TRIM6 degradation resulting in reduced synthesis of K48-linked unanchored polyubiquitin chains, reduced IKKε oligomerization, IKKε-T501 autophosphorylation and reduced IRF3 phosphorylation with impaired antiviral responses.

Taken together, our data shows that NiV matrix protein inhibits IFN responses by targeting the E3-ubiquitin ligase TRIM6 during infection or ectopic expression in cell lines and primary innate immune cells. Reduction in TRIM6 expression correlates with reduced unanchored polyubiquitin chains that specifically associate with IKKε and have been previously shown to be required for IKKε activation, ultimately resulting in impaired induction of IFN-mediated antiviral responses (Fig 8).

## Discussion

In this study we demonstrate that the matrix protein of NiV inhibits the antiviral IFN system by targeting the E3-ubiquitin ligase TRIM6, which is required for the synthesis of unanchored polyubiquitin chains that activate the IKKε kinase for IFN production. Our findings indicate that the NiV-M protein plays a role in the context of virus replication and in primary innate immune cells. This is supported by three lines of evidence: i) lentiviruses expressing NiV-M strongly inhibited SeV-induced IFNβ induction but not pro-inflammatory cytokines in hMDDC, ii) infections with WT NiV reduce expression of TRIM6 protein and the formation of TRIM6-cytoplasmic bodies, iii) infections with a recombinant NiV lacking M protein rescue TRIM6 protein levels.

We have shown that a K258A mutation of NiV-M, which is impaired in cytoplasm-nuclear trafficking ([38] and Fig 5D), has reduced ability to inhibit IFN-I responses. In contrast, the mutants L10-K258A-M and S15-K258A-M, which recover trafficking to cytoplasmic membrane fractions [38], recovered the ability to antagonize IFN-I responses (Fig 3B). Since NiV-M-K258A can still interact with TRIM6 in coIP and colocalization studies and is able to compete with IKKε for TRIM6 binding, our data suggest that the reduced ability of M-K258A to inhibit IFN-I is due to cellular mislocalization, resulting in failure to target TRIM6 for degradation. Most likely, the K258 amino acid on NiV-M is not required for interaction with TRIM6, and is instead required for targeting M protein to the cytoplasmic compartment where the TRIM6—IKKε “signalosome” assembles, and NiV-M may recruit other factors required for TRIM6 degradation. We previously showed that TRIM6 recruits IKKε to “ubiquitin-rich” bodies in the cytoplasm and that unanchored polyubiquitin is important for the formation of these structures [13]. Although these TRIM6-ubiquitin-rich structures have not been well characterized, it is unlikely that they contain membranous structures. Some studies on TRIM5α, a close relative of TRIM6, suggest that some of these cytoplasmic bodies may contain components of the aggresome system [53, 54]. An alternative possibility is that ubiquitination of NiV-M on the K258 amino acid, which has been shown to be important for trafficking of M [38] may also be responsible for recruiting other factors involved in TRIM6 degradation. These possibilities are currently under investigation.

Our results show that NiV-M targets TRIM6 for degradation. However, we have so far not been able to elucidate the precise pathway involved in TRIM6 degradation. Experiments using the proteasome inhibitor MG132 [55] and the lysosome inhibitor chloroquine [56] did not appear to rescue endogenous levels of TRIM6 protein in the presence of NiV-M (Fig 6B and S8 Fig), suggesting that NiV-M targets TRIM6 for degradation by a proteasome- and lysosome-independent mechanism. We are currently investigating potential pathways of TRIM6 degradation using other inhibitors that could potentially recover IFN responses by blocking TRIM6 degradation. If successful, inhibitors that rescue TRIM6 expression and IFN responses may have potential clinical use as antivirals to NiV infections.

Importantly, NiV-M not only impaired TRIM6-mediated IFN induction upon stimulation of the RIG-I pathway in primary human DCs and in cell lines, but also impaired IFNβ signaling. These results are in line with the multiple roles described for IKKε and TRIM6 in both the IFN induction and signaling pathways [8, 13, 14].

Our data indicates that NiV-M can inhibit IFN induced by both TBK-1 and IKKε when ectopically expressed. However, in our previous studies we did not find interaction of TRIM6 with TBK-1 or effects on TBK-1 activation in TRIM6 knockdown cells, suggesting that the mechanisms by which NiV-M inhibits TBK-1 and IKKε are different. One possibility is that NiV-M is able to inhibit other TRIM proteins responsible for TBK-1 activation.

Henipaviruses are zoonotic pathogens that have been found in Asian fruit bats of the Pteropus genus, without causing evident disease. Henipaviruses have also been reported to inhibit innate immune pathways in bats to establish infections in their natural reservoir [35]. Interestingly, TRIM6 is conserved across mammalian species including bats of the genus Pteropus (S5 Fig). Human TRIM6 shares relatively high homology with bat TRIM6 (80–84% amino acid identity, S6 Fig). Importantly the cysteine and histidine residues in the RING domain, which are important for TRIM6 E3-ubiquitin ligase activity, are conserved between species, suggesting that TRIM6 is active in bats. The C-terminal SPRY domain of human TRIM6, which is responsible for M interaction, also shares 80–84% homology. Although it is difficult to predict whether NiV-M is still able to interact and inhibit TRIM6-medaited antiviral responses in bats, it is tempting to speculate that M proteins of henipaviruses may also be able to antagonize IFN responses in the bats, especially because other factors of the RIG-I pathway and specifically TBK1 and IKKε (97% and 80% identity respectively), are also relatively conserved in bat species (S7 Fig). Therefore, TRIM6 and the IKKε signalosome may be highly relevant in antiviral responses in the natural reservoir of henipaviruses.

The matrix protein is a viral structural protein with functions in virus assembly and budding. Our findings have important implications on the efficiency of NiV replication. The fact that the matrix protein is contained in the virion and is released immediately upon virus entry gives the virus a tool to fight the host antiviral response early during virus replication and before the IFN response is activated, giving an advantage to the virus. Pharmacological approaches could be developed to target NiV-M interactions with TRIM6 or the use of inhibitors to block TRIM6 degradation promoted by M.

In summary, here we report an additional paramyxovirus protein with IFN antagonist function. The matrix protein of Nipah virus inhibits both the IFN induction and the IFN signaling pathways by promoting degradation of the E3-ubiquitin ligase TRIM6, which synthesizes unanchored polyubiquitin chains required for IKKε activation and induction of an efficient antiviral response (Fig 8). This is the first example of a structural protein of henipaviruses with IFN antagonist functions.

## Materials and Methods

### Cells and viruses

HEK-293T, HeLa, A549, Vero E6 (CRL1586), and Vero (CCL-81) cells were all purchased from the American Type Culture Collection (ATCC). All cells were maintained in Dulbecco's Modified Eagle's Medium (DMEM) supplemented with 10% fetal bovine serum (FBS), 2 mM L-glutamine and 1% penicillin-streptomycin (Gibco-BRL). Sendai virus (SeV; Cantell strain) was obtained from Charles River Laboratories and propagated in 10-day old pathogen-free embryonated chicken eggs (Charles River Laboratories; North Franklin, CT). For infection, 293T cells were incubated with SeV for 2hrs at 37°C, and then the medium was changed to complete growth medium. The Nipah virus (NiV) strain Malaysia (kindly provided by the Special Pathogens Branch, CDC, Atlanta) was propagated in VeroE6 cells. Stock virus titer was determined by plaque assay in Vero-CCL81 cells. For infection, confluent monolayers of 293T cells (seeded in 24-well-plates) were infected with NiV (MOI 0.01 to 1.0) for 1 hr at 37°C, and then fresh medium containing 2% FBS, 100 U/mL penicillin and 100 μg/mL streptomycin was added. Cells were harvested at 24 h.p.i. in 100μl PBS, lysed in 6x SDS-PAGE Laemmli buffer and incubated for 15 min at 95°C. All work with live virus was carried out under Biosafety Level 4 (BSL4) conditions in the Robert E. Shope BSL4 Laboratory, UTMB.

### Plasmids and reagents

The NiV-M, NiV-K258A, K258R, 3XFlag-tagged NiV-M and mutant K258A expression constructs have been described before [38]. The NiV-V expression construct was made by PCR amplification of the NiV-V open reading frame (ORF) and insertion into the pcDNA3.1(+) vector with an N-terminal HA tag. The TRIF, MAVS, TBK-1 and IKKε expression constructs as well as pIFNβ_fLuc, ISRE_fLuc reporters were kindly provided by Dr. Genhong Cheng and have been described previously. The IRF3 expression construct was provided by Dr. Ren Sun. Reporter plasmids expressing firefly luciferase under the control of the ISG54-ISRE and the IFNβ promoter were described previously [4, 57], and were a generous gift from Dr Garcia-Sastre (Mount Sinai, NY). FLAG-tagged RIG-I(2CARD) was previously described [58]. The reporter plasmid carrying the Renilla luciferase gene (REN-Luc/pRL-TK) was purchased from Promega. The HA-TRIM6 plasmid was kindly provided by Andrea Ballabio [51]. All sequences were confirmed by sequencing analysis (Genewiz, NJ) and at the UTMB molecular genomics core facility.

Transient transfections were performed with TransIT-LT1 (Mirus), Lipofectamine 2000 or RNAiMax (Invitrogen) according to the manufacturers' instructions. Rabbit anti-NiV-M antibody has been described previously [38]. Rabbit anti-phospho-IRF3 (Ser396) antibody (4D4G) and mouse anti-IRF3 (3F10) antibody were from Cell Signaling and Immuno-Biological Laboratories, respectively. Rabbit anti TRIM6 (N term) antibody, rabbit and mouse anti c-myc antibody, mouse and rabbit anti-FLAG antibodies, rabbit anti-HA antibody, mouse anti-β-tubulin and mouse anti-β-actin antibodies were from Sigma. Rabbit anti phospho IKKε antibody (T501) was purchased from Novus Biologicals. Rabbit monoclonal anti-ubiquitin Lysine 48 (K48, clone Apu2) was purchased from Millipore. Rabbit anti GST antibody (OTI4G1) was from Bethyl Laboratories. Fluorescently labeled secondary antibodies for imaging: Alexa Fluor 488 goat anti mouse, Alexa Fluor 488 donkey anti rabbit, Alexa Fluor 555 goat anti mouse, Alexa Fluor 555 donkey anti rabbit, goat anti mouse Alexa Fluor 633, were purchased from ThermoScientific (Life Technologies). Directly conjugated antibodies towards Flag/DYKDDDK tag rabbit (Alexa 555) and HA tag mouse (Alexa 488) were from ThermoScientific and Cell Signaling Technologies.

### Luciferase reporter assays

HEK293T cells were transfected in 24 (50X10^3^ cells per well) or 96-well plates (10X10^3^ cells per well) (Falcon, Becton Dickinson, NJ) with 10–50 ng of IFNβ/ISG54 ISRE reporter plasmid, 4–20 ng of Renilla luciferase and 2–50 ng plasmids using TransIT-LT1 (Mirus), at a ratio 1:3. Empty vector was used to ensure that the plasmid concentration in each well was the same. 24 h later, cells were lysed and dual-luciferase assay was performed according to the manufacturer's instructions (Promega, Madison, WI, USA). Percentage inhibition was calculated first by normalizing the Luciferase values by Renilla values (fLuc/rLuc). Then, in each graph the positive control which has an activator (SeV, IFNβ, TRIF etc.) but no inhibitor (i.e. NiV-M or NiV-V) was set to 100% and everything else was normalized to this control sample.

### Co-immunoprecipitation and western blotting

Transfected 293T cells were harvested in RIPA lysis buffer containing 50 mM TRIS, pH8.0, 280 mM NaCl, 0.5% [v/v] NP40, Glycerol 10%, protease inhibitor cocktail [Roche and supplemented with 5 mM N-ethylmaleimide (NEM) and Iodoacetamide as deubiquitinase inhibitors. Cell lysate was clarified by microcentrifugation at 14,000rpmi for 20 min. One tenth of the aliquot from clarified lysate was taken and added to 2x Laemmli buffer with β-ME and stored at -20°C for western blots (Whole cell extracts, [WCE]). To the rest of the lysate mouse anti-FLAG or anti-HA antibody cross-linked to agarose beads (EZ View Red Anti-FLAG M2 or EZ View Red Anti-HA Affinity Gel Sigma) were added and rotated on a bench top shaker overnight at 4°C. The next day, beads were extensively washed, and the bound proteins were eluted by boiling for 10 min in Laemmli loading buffer.

For immunoblotting, proteins were resolved by SDS-polyacrylamide gel electrophoresis (7.5% or 4–15% SDS-PAGE) and transferred onto a PVDF membrane (Immobilon-P Millipore or BioRad Laboratories). The following primary antibodies were used: anti-Ub-K48 (1:1,000), anti-Flag (1:3,000) (Sigma), anti-HA (1:5,000) (Sigma), anti-GST (1:2,000) (Bethyl Laboratories), anti-myc (1:2,000) (Sigma), anti-NiV M (1:3000), anti TRIM6 N term (1:1000), pIKKε (1:500), anti pIRF3 (1:1,000), anti IRF3 (1:3000), anti actin/tubulin (1:5000).

Immunoblots were developed with the following secondary antibodies: ECL anti-rabbit IgG horseradish peroxidase conjugated whole antibody from donkey, and ECL anti-mouse IgG horseradish peroxidase conjugated whole antibody from sheep (GE Healthcare; Buckinghamshire, England). The proteins were visualized by an enhanced chemiluminescence reagent (Pierce).

### siRNA-mediated gene targeting

Transient knockdown of endogenous TRIM6 in human 293T cells, seeded in 96-well plates, was achieved as described before [13]. Briefly, by transfection of 10 pmol of non-targeting control or an siRNA specific for TRIM6 (Life technologies, sleath RNAi TRIM6-specific sequence targeting the 5’-UTR region of transcript variant 2; sense: GCUGCUUCAAGUCCUUGGCUCUGAU and antisense: AUCAGAGCCAAGGACUUGAAGCAGC), with RNAiMAX (Invitrogen) according to the manufacturer’s instructions. Rescue of knockdown was achieved by transfecting TRIM6 encoding plasmid using TransIT-LT1 (Mirus) 24h post silencing. Because the siRNA targets the untranslated region of TRIM6, these siRNA sequences do not attenuate TRIM6 expression from the expression vector upon transfection. TRIM6 knockdown and rescue efficiency were determined by western blotting and real time PCR using specific primers as described before [13].

### Quantitative PCR

QPCR was done as previously described [44]. In brief, total RNA was extracted from hMDDCs with TRIzol reagent (Sigma). cDNA was prepared by using a SuperScript III first-strand synthesis system (Invitrogen). Relative gene expression was determined by using PerfeCTa SYBR green FastMix (Quanta Biosciences, Inc.) with a Bio-Rad CFX96 instrument. CXF Manager software (Bio-Rad) was used to analyze the relative mRNA expression levels by the change in the threshold cycle (ΔCT), with the RPS11 gene serving as a reference mRNA to which the results were normalized. The copy number for RPS11 was based upon a standard curve generated by using an RPS11-containing plasmid.

### Immunofluorescence microscopy and image analysis

For colocalization studies of TRIM6 and NiV-M or mutants, HeLa cells were seeded into Lab-Tek II 8-well chamber slides (CC2 Glass slide, Nunc; Rochester, NY). After 12–16 h, 300-700ng of plasmids harboring NiV-M, NiV-K258A, NiV-K258R, HA-TRIM6 or empty vector backbone were transfected with Lipofectamine 2000 (Invitrogen) at a ratio 1:1. Six hours later, media was replaced and 16–24 h later, cells were washed with PBS, fixed with 4% paraformaldehyde, permeabilized with 0.5% NP-40 (v/v) in PBS, and blocked with 0.5% BSA 0.2% fish gelatin in PBS for 1h (blocking solution). For NiV-M or its mutants, cells were stained with primary rabbit anti-M antibody (1:1000) [38] and for HA-TRIM6, anti-HA antibody (1:200) Alexa Fluor 488 (prepared in blocking solution) overnight at 4°C. The next day, cells were washed 3 times with blocking solution and secondary antibody donkey anti-rabbit Alexa-Fluor 555 (Invitrogen) diluted in blocking buffer along with DAPI (1:2000) were used to visualize the proteins. The slides were imaged on a Zeiss LSM 510 confocal microscope in the UTMB optical imaging core at a magnification of 63×. For non-confocal imaging, slides were observed and imaging was done on Bio-Tek Cytation5 plate reader with fluorescence microscope.

For the live viral infection experiments, cells were infected with recombinant Nipah virus expressing EGFP (rNiV-EGFP^NP^) [59] (depicted in the text as NiV-WT) or rNiV-EGFP^NP^ lacking the matrix protein (rNiV-EGFP^NP^ delta M, depicted in the text as NiV-ΔM) at an MOI of 0.1. 24-48h post infection, cells were fixed with 10% formalin for 24 hr and removed from the BSL4. Cells were washed extensively with PBS, permeabilized with 0.5% NP-40 (v/v) in PBS, and blocked with 0.5% BSA 0.2% fish gelatin in PBS for 1h (blocking solution).

Cells infected with rNiV-EGFP^NP^ or rNiV-EGFP^NP^ delta M were stained for TRIM6 using rabbit anti-TRIM6 N-term antibody (Sigma) (1:200) overnight at 4°C, washed 3X with blocking buffer the next day and stained with secondary donkey anti rabbit Alexa Fluor 555 (1:500) and counterstained with DAPI (1:2000) for 1h (Fig 7A and 7B). Alternatively, cells were stained for anti-NiV-M antibody [38] (1:1000) along with mouse anti TRIM6 antibody (1:100) overnight at 4°C followed by three washes the next day. Cells were then stained with secondary donkey anti rabbit Alexa Fluor 555 (1:500) and goat anti-mouse Alexa Fluor 633 and counterstained with DAPI (1:2000). After extensive washing with PBS, cells were mounted using Vecta shield mounting medium and imaged on a Zeiss LSM 510 confocal microscope in the UTMB optical imaging core and Bio-Tek cytation5 (Fig 7C).

Cells were manually counted, included or excluded by inspection to ensure that all cells included in the final scoring had the cell boundary correctly defined. ImageJ software was used to calculate ratio between NiV-M and TRIM6 mean fluorescence intensities (MFI). For this quantification, individual cells were selected using the freehand selection tool in imageJ software following the borders of each individual cell based on TRIM6 staining. Over 50 cells were selected for quantification in ImageJ software. A minimum cutoff intensity level was applied to ensure NiV-M expression was sufficient.

### Rescue of recombinant NiV rNiV-EGFP^NP^ delta M virus (NiV-ΔM)

The T7-driven rNiV-ΔM rescue construct was derived from rNiV with firefly luciferase between the N and P genes [59]. The M gene was deleted via replacement by EGFP. HEK293T cells were transfected with helper plasmid encoding NiV-M (0.5μg) to facilitate budding of mature virions. Four hours later, cells were transfected with helper plasmids encoding codon-optimized T7 polymerase (1μg), NiV-N (1μg), NiV-P (0.8μg), NiV-L (0.2μg), and full-length rNiV-ΔM (3.5μg) using TransIT-LT1 reagent. Supernatants were collected on day 5, and rescued virus was then propagated through infections of NiV-M-inducible 293-pTRE3G-M cells. To generate these cells, codon-optimized NiV-M in pTRE3G (Clontech) was introduced into HEK293 Tet-On 3G cells (Clontech) and single cell-cloned for doxycycline-inducible expression of NiV-M. Propagation in G418 maintained the Tet-on transactivator, and hygromycin maintained pTRE3G-NiV-M, which was stably integrated along with a linear hygromycin marker (via co-transfection). M expression was induced by the addition of 30ng/ml doxycycline prior to infection with rescued rNiV-ΔM. Supernatant was then collected 5 days post-infection. Virus titers were determined by standard plaque assays in Vero cells. The lack of M expression was confirmed by Western blot. (S4 Fig).

### Isolation of human monocyte-derived DCs (hMDDC) and lentiviral transduction

Human MDDCs were generated from CD14^+^ cells purified from concentrated leukocytes of healthy human donors (New York Blood Center), as described previously [42, 44]. Briefly, peripheral blood mononuclear cells (PBMCs) were isolated by Ficoll density gradient centrifugation and CD14^+^ cells were purified by using CD14 microbeads. CD14^+^ cells, were incubated at 37°C for 5 days in DC medium (RPMI containing 4% human AB serum, 2 mM L-glutamine, 1 mM sodium pyruvate, 100 U/ml penicillin–100 μg/ml streptomycin, and 55 μM β-mercaptoethanol) supplemented with 500 U/ml human granulocyte-macrophage colony-stimulating factor and 500 U/ml human interleukin-4 (hIL-4; PeproTech). MDDCs were transduced by spinoculation at 1,850 rpm with lentiviruses and Vpx-VLPs for 2.5 h and then cultured in fresh medium for 72 h. Transduced MDDCs were harvested to assess expression by flow cytometry and Western blotting or were used in subsequent experiments.

### Ethics statement

The leukocytes from healthy human donors were obtained from the New York Blood Center. These samples are anonymous blood bank donor samples. This constitutes exempt research and does not require IRB review.

### Statistical analysis

Statistical analysis was performed with Prism (Version 5.0, GraphPad Software) using Student’s paired t test or in defined cases two-way ANOVA with Bonferroni post-test were used. *p < 0.05; **p < 0.01; ***p < 0.001, ****p < 0.0001

## Supporting Information

S1 Fig Nipah virus matrix protein inhibits IFNβ mRNA induction in SeV-infected cells and increased SeV replication. HEK293T cells were transfected with NiV-M or empty vector for 30 hr followed by SeV infection. Cells were lysed at different time points p.i. for RNA extraction and qPCR analysis.
(TIFF)

S2 Fig Quantification of immunoblots shown in Fig 3C (left column) and 3E (right column). Quantification was performed using ImageJ software and values were normalized by the levels of immunoprecipitated IKKε.
(TIF)

S3 Fig NiV-M-WT and K258A interact with TRIM6 and compete for TRIM6 interaction with IKKε. A) HEK293T cells were transfected with NiV-M-WT or NiV-M-K258A, empty vector or HA-TRIM6 and IKKε. Cells were harvested and whole cell extracts (WCE) were used for TRIM6 immunoprecipitation using anti-HA-beads (A), or for the reverse coIP for IKKε by using anti-Flag beads (B).
(TIFF)

S4 Fig NiV-ΔM virus has delayed growth kinetics. A) rNiV-WT and ΔM growth kinetics in Vero cells at a starting MOI of 0.01. B) rNiV-ΔM does not express matrix protein. Samples were collected at each time point for immunoblot.
(TIFF)

S5 Fig TRIM6 is conserved across mammalian species. Phylogenetic analysis of TRIM6. Multiple protein sequence alignment was performed using all the TRIM6 sequences reported in the NCBI website. The results of the sequence alignment was used to build a joint-neighboring phylogenetic Tree using the NCBI website.
(TIF)

S6 Fig Human TRIM6 protein shares high degree of homology with bat TRIM6. Multiple protein sequence alignment was performed using human and bat TRIM6 sequences reported in the NCBI website.
(TIF)

S7 Fig Factors of the RIG-I/MDA5 pathway shares high degree of homology between humans and bats. Multiple protein sequence alignment was performed using human and bat protein sequences reported in the NCBI website and amino acid identity is shown.
(TIFF)

S8 Fig HEK293T cells were transfected with NiV-M-WT or empty vector for 24 hours. Cells were then treated with Chloroquine overnight. Detection of endogenous TRIM6 is shown by immunoblot.
(TIFF)
