# Supplementary figures and images for: The Matrix Protein of Nipah Virus Targets the E3-Ubiquitin Ligase TRIM6 to Inhibit the IKKε Kinase-Mediated Type-I IFN Antiviral Response
Source: PLoS Pathog. 2016 Sep 13;12(9):e1005880. doi: 10.1371/journal.ppat.1005880 (PMC5021333; doi:10.1371/journal.ppat.1005880)

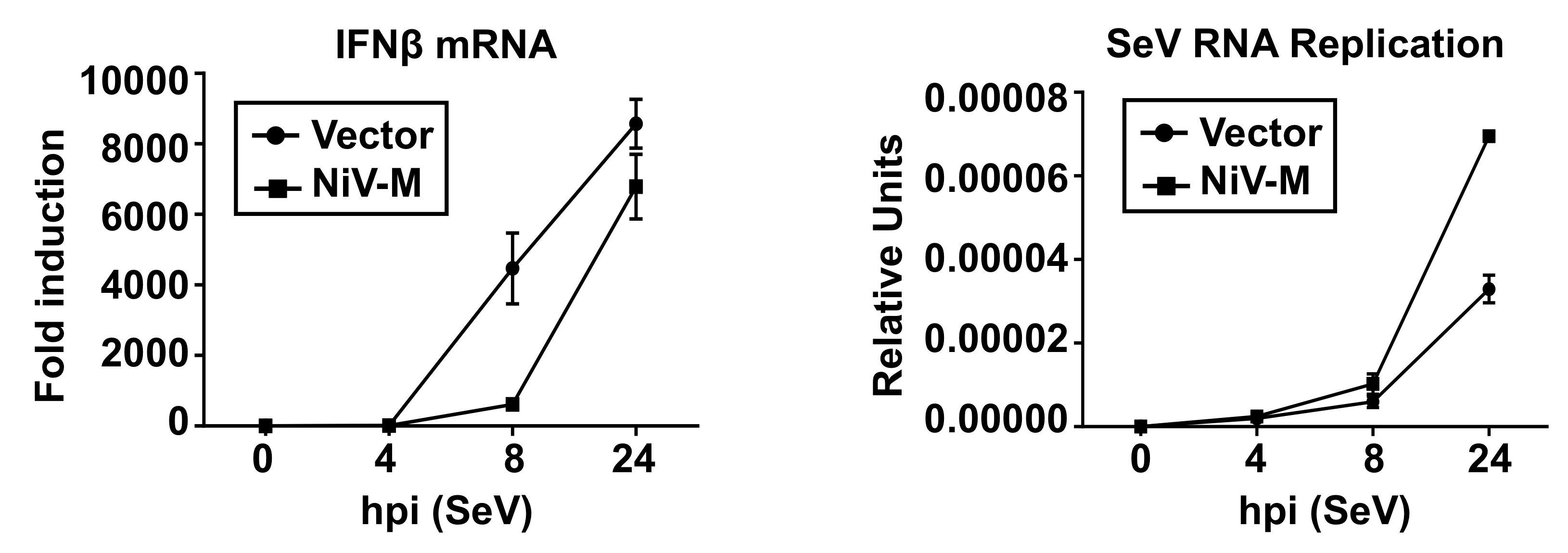

Supplement: S1 Fig — HEK293T cells were transfected with NiV-M or empty vector for 30 hr followed by SeV infection. Cells were lysed at different time points p.i. for RNA extraction and qPCR analysis. (TIFF) [file ppat.1005880.s001.tiff]

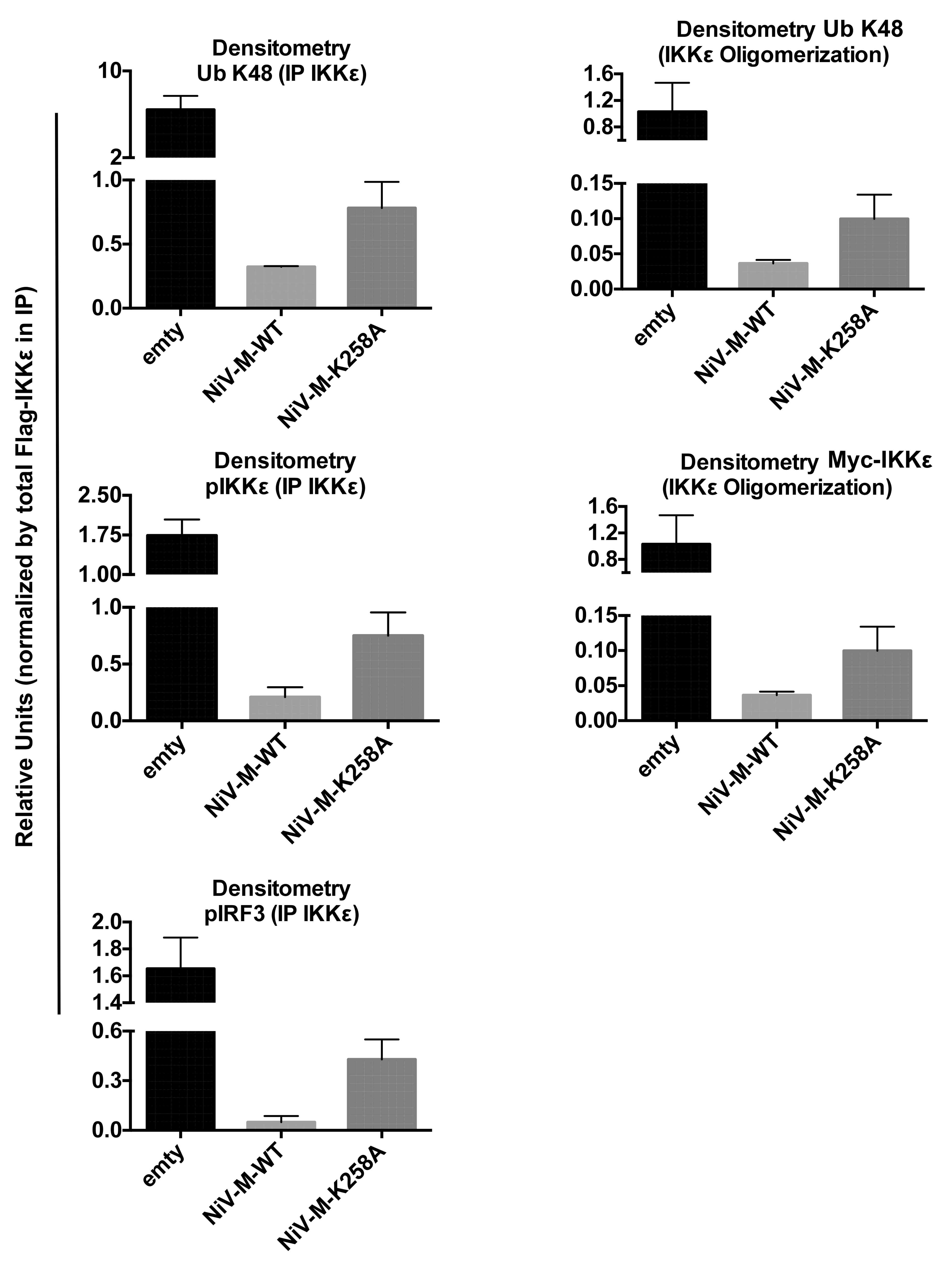

Supplement: S2 Fig — Quantification was performed using ImageJ software and values were normalized by the levels of immunoprecipitated IKKε. (TIF) [file ppat.1005880.s002.tif]

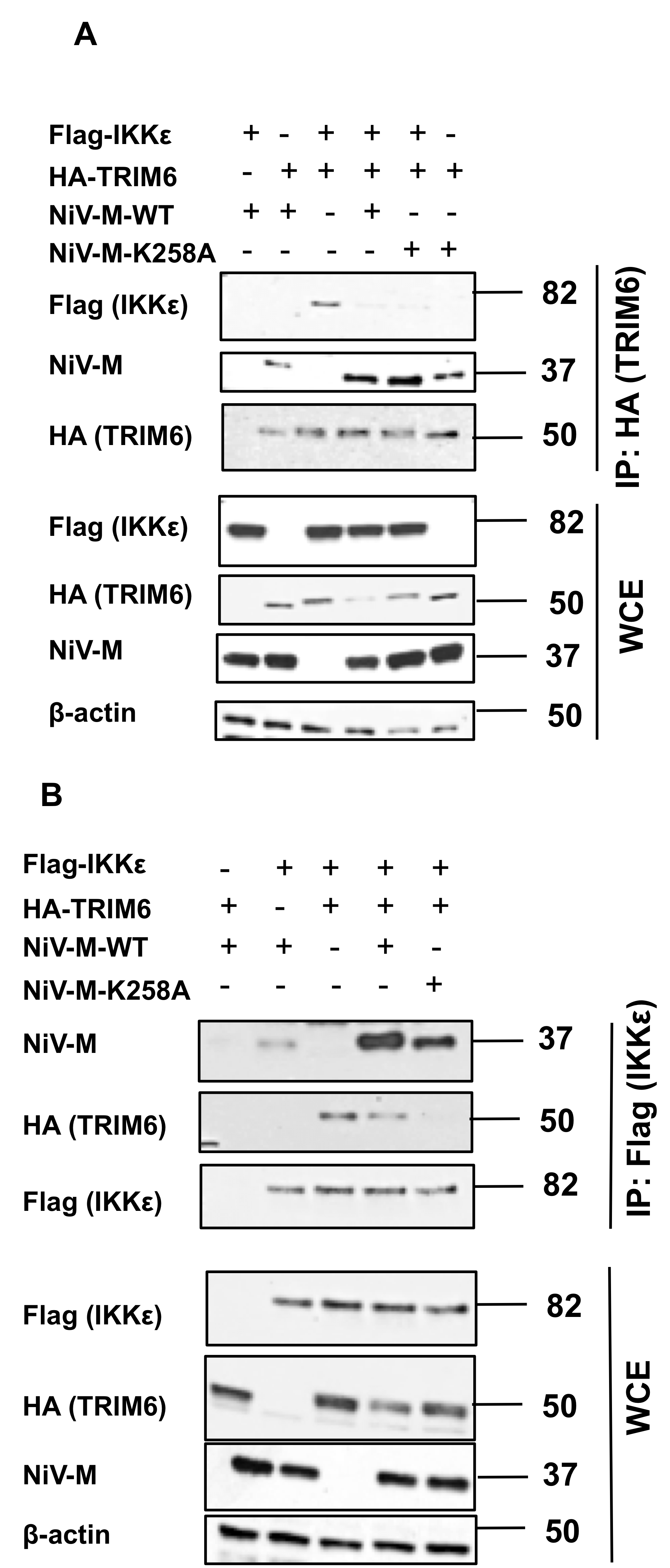

Supplement: S3 Fig — A) HEK293T cells were transfected with NiV-M-WT or NiV-M-K258A, empty vector or HA-TRIM6 and IKKε. Cells were harvested and whole cell extracts (WCE) were used for TRIM6 immunoprecipitation using anti-HA-beads (A), or for the reverse coIP for IKKε by using anti-Flag beads (B). (TIFF) [file ppat.1005880.s003.tiff]

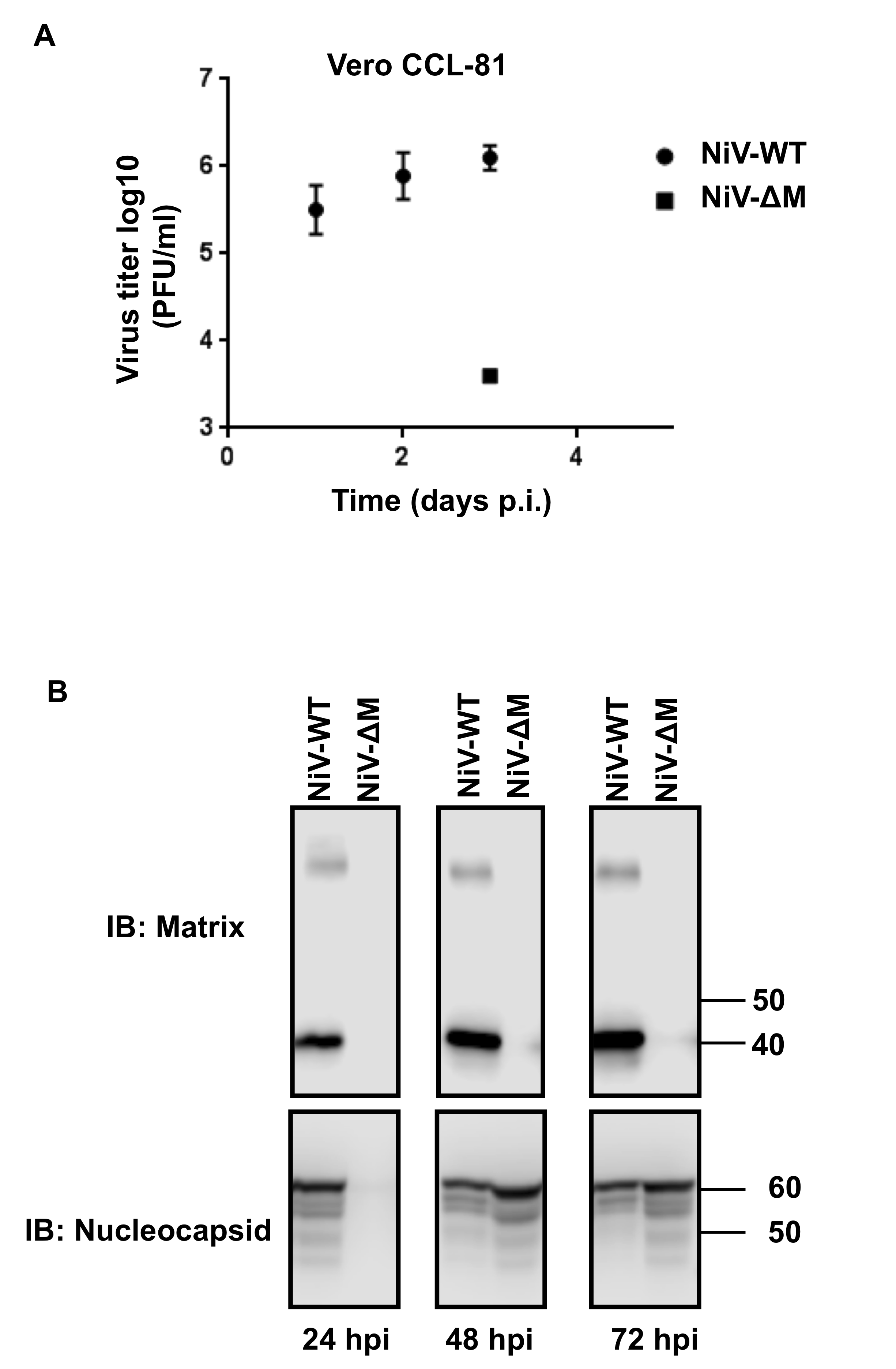

Supplement: S4 Fig — A) rNiV-WT and ΔM growth kinetics in Vero cells at a starting MOI of 0.01. B) rNiV-ΔM does not express matrix protein. Samples were collected at each time point for immunoblot. (TIFF) [file ppat.1005880.s004.tiff]

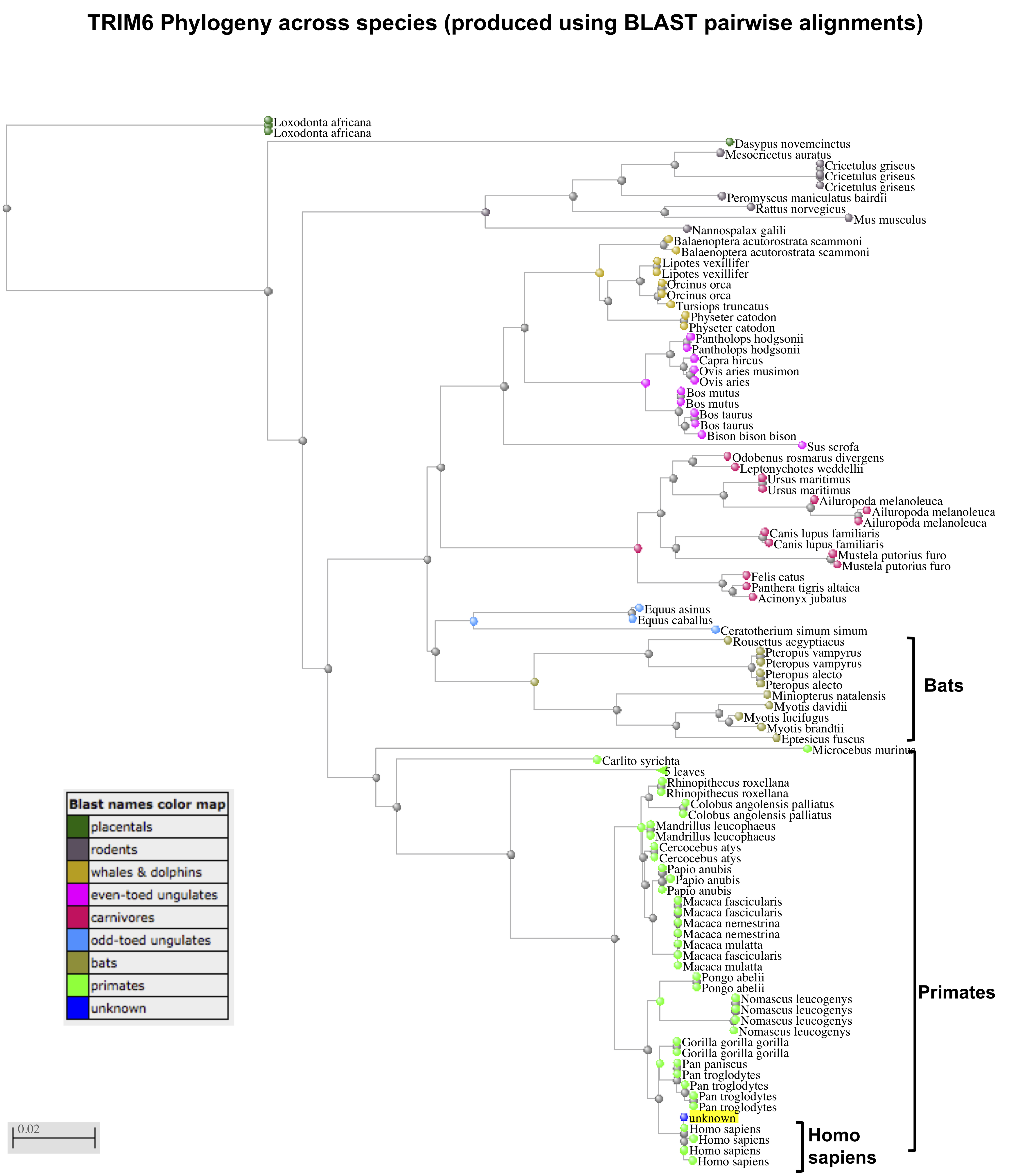

Supplement: S5 Fig — Phylogenetic analysis of TRIM6. Multiple protein sequence alignment was performed using all the TRIM6 sequences reported in the NCBI website. The results of the sequence alignment was used to build a joint-neighboring phylogenetic Tree using the NCBI website. (TIF) [file ppat.1005880.s005.tif]

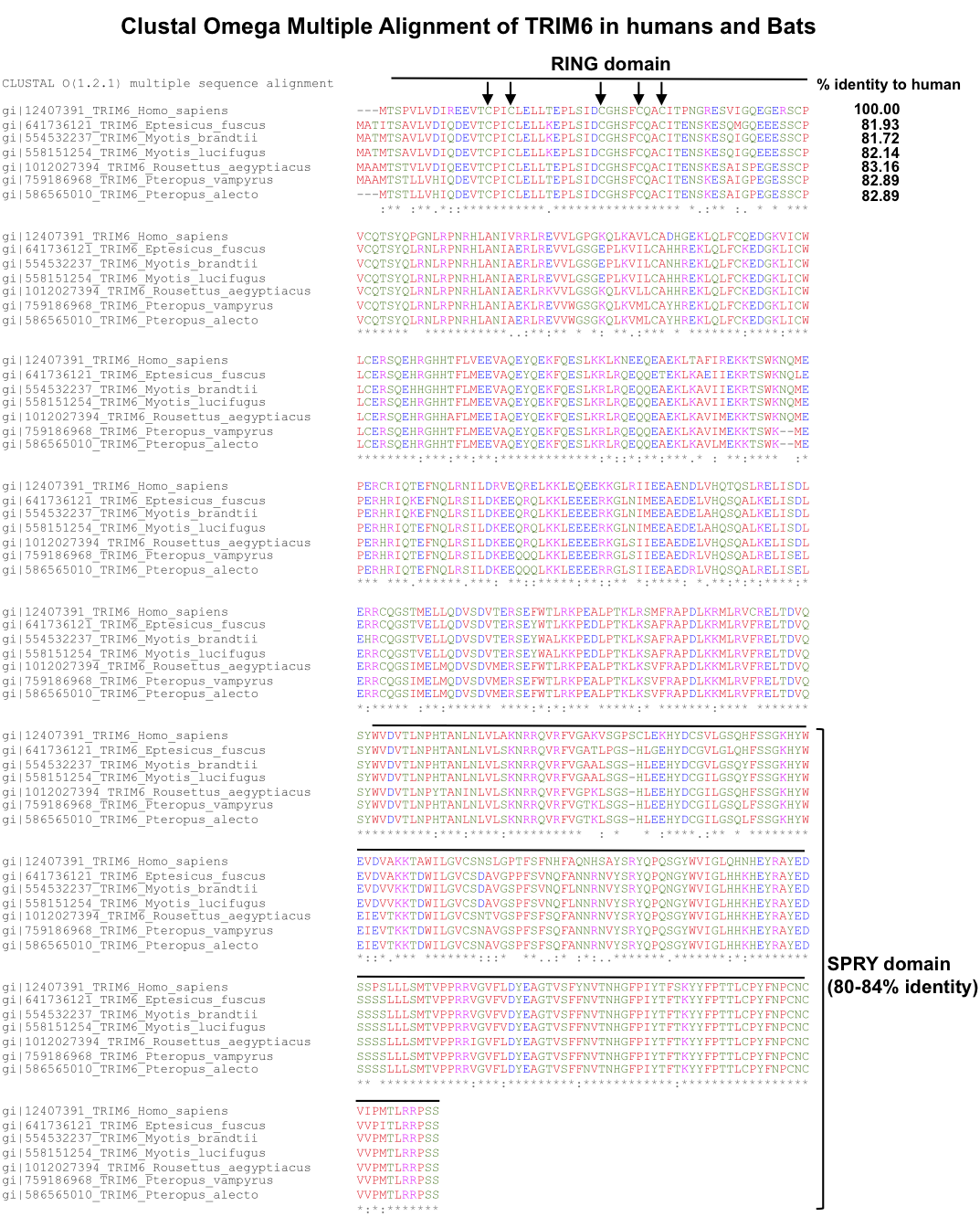

Supplement: S6 Fig — Multiple protein sequence alignment was performed using human and bat TRIM6 sequences reported in the NCBI website. (TIF) [file ppat.1005880.s006.tif]

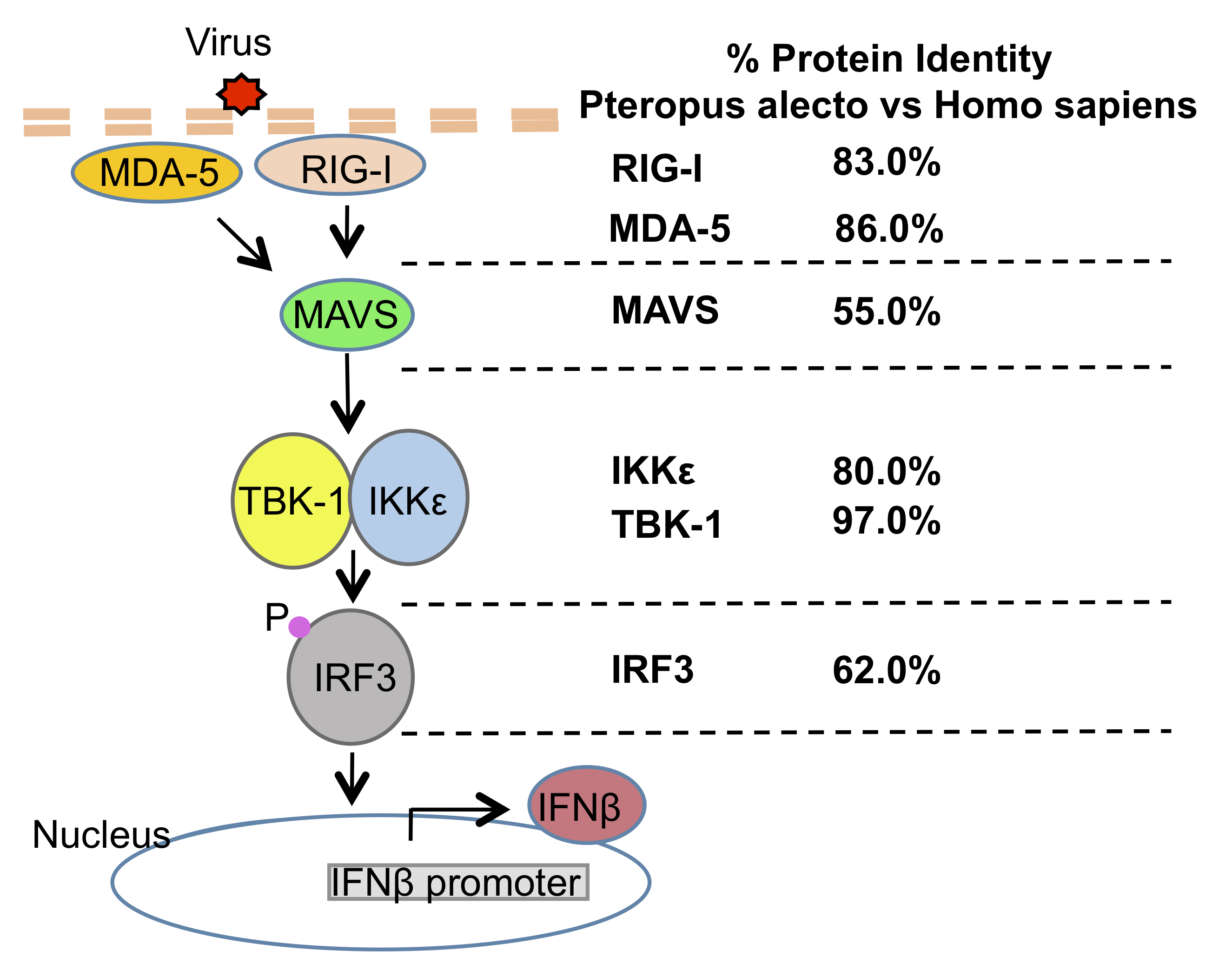

Supplement: S7 Fig — Multiple protein sequence alignment was performed using human and bat protein sequences reported in the NCBI website and amino acid identity is shown. (TIFF) [file ppat.1005880.s007.tiff]

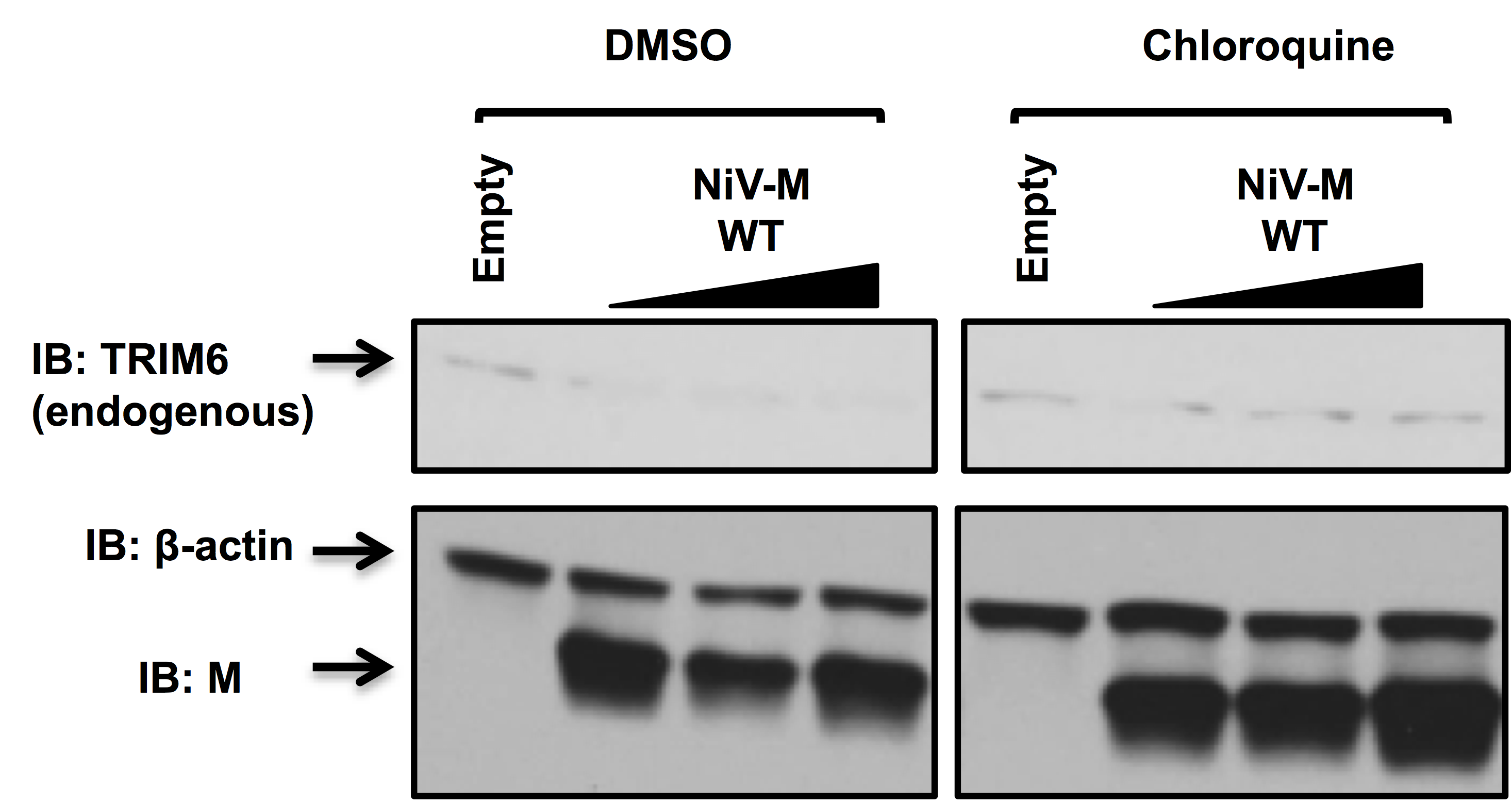

Supplement: S8 Fig — Cells were then treated with Chloroquine overnight. Detection of endogenous TRIM6 is shown by immunoblot. (TIFF) [file ppat.1005880.s008.tiff]
